# A positive feedback loop between PFKP and c-Myc drives head and neck squamous cell carcinoma progression

**DOI:** 10.1186/s12943-024-02051-6

**Published:** 2024-07-09

**Authors:** Weiwei Liu, Zhao Ding, Ye Tao, Shixian Liu, Maoyu Jiang, Fangzheng Yi, Zixi Wang, Yanxun Han, Huaiyuan Zong, Dapeng Li, Yue Zhu, Zihui Xie, Shujia Sang, Xixi Chen, Manli Miao, Xu Chen, Wei Lin, Yi Zhao, Guibin Zheng, Mark Zafereo, Guojun Li, Jing Wu, Xiaojun Zha, Yehai Liu

**Affiliations:** 1 Department of Otolaryngology, Head & Neck Surgery, The First Affiliated Hospital of Anhui Medical University, Hefei, 230022 China; 2 Department of Biochemistry & Molecular Biology, School of Basic Medicine, Anhui Medical University, No. 81 Meishan Road, Hefei, Anhui Province 230032 China; 3 Department of Otolaryngology, Head & Neck Surgery, The Affiliated Bozhou Hospital of Anhui Medical University, Bozhou, 236800 China; 4 Department of Oncology, the First Affiliated Hospital of Anhui Medical University, Hefei, 230022 China; 5 Department of Stomatology, the First Affiliated Hospital of Anhui Medical University, Hefei, 230022 China; 6 Institutes of Biomedical Sciences, Children’s Hospital of Fudan University, National Children’s Medical Center, Fudan University, Shanghai, 200032 China; 7 Department of Thyroid Surgery, the Affiliated Yantai Yuhuangding Hospital, Qingdao University, Yantai, Shandong 264000 China; 8 Department of Head and Neck Surgery, The University of Texas MD Anderson Cancer Center, Houston, TX 77030 USA

**Keywords:** PFKP, c-Myc, ERK, HNSCC, Tumor progression

## Abstract

**Background:**

The aberrant expression of phosphofructokinase-platelet (PFKP) plays a crucial role in the development of various human cancers by modifying diverse biological functions. However, the precise molecular mechanisms underlying the role of PFKP in head and neck squamous cell carcinoma (HNSCC) are not fully elucidated.

**Methods:**

We assessed the expression levels of PFKP and c-Myc in tumor and adjacent normal tissues from 120 HNSCC patients. A series of in vitro and in vivo experiments were performed to explore the impact of the feedback loop between PFKP and c-Myc on HNSCC progression. Additionally, we explored the therapeutic effects of targeting PFKP and c-Myc in HNSCC using Patient-Derived Organoids (PDO), Cell Line-Derived Xenografts, and Patients-Derived Xenografts.

**Results:**

Our findings indicated that PFKP is frequently upregulated in HNSCC tissues and cell lines, correlating with poor prognosis. Our in vitro and in vivo experiments demonstrate that elevated PFKP facilitates cell proliferation, angiogenesis, and metastasis in HNSCC. Mechanistically, PFKP increases the ERK-mediated stability of c-Myc, thereby driving progression of HNSCC. Moreover, c-Myc stimulates PFKP expression at the transcriptional level, thus forming a positive feedback loop between PFKP and c-Myc. Additionally, our multiple models demonstrate that co-targeting PFKP and c-Myc triggers synergistic anti-tumor effects in HNSCC.

**Conclusion:**

Our study demonstrates the critical role of the PFKP/c-Myc positive feedback loop in driving HNSCC progression and suggests that simultaneously targeting PFKP and c-Myc may be a novel and effective therapeutic strategy for HNSCC.

**Supplementary Information:**

The online version contains supplementary material available at 10.1186/s12943-024-02051-6.

## Introduction

Head and neck squamous cell carcinoma (HNSCC) ranks at the sixth most common cancer worldwide [1, 2]. It typically originates from squamous cells lining the mucosal surfaces in the head and neck region. These cancers are categorized based on their anatomical location, which includes the oral cavity, pharynx (nasopharynx, oropharynx, hypopharynx), larynx, paranasal sinuses, nasal cavity, and salivary glands [3]. Due to their relatively concealed anatomical location, most HNSCC cases are diagnosed at an advanced stage [4]. Although the treatment of HNSCC is multimodal, including surgery, radiotherapy, chemotherapy, targeted therapy, and immunotherapy [5], the overall survival (OS) rate for HNSCC patients has not improved over the past decade [6]. The lack of accessible targeted therapies and inadequate clinical management underscore the necessity for research into the molecular mechanisms underlying HNSCC pathogenesis.

Cancer cells typically exhibit distinctive metabolic alterations, relying on glycolysis for energy production, leading to lactic acid fermentation in the cytosol, even when oxygen is available. In the glycolytic pathway, phosphofructokinase 1 (PFK-1) plays a pivotal role as the rate-limiting enzyme catalyzing the conversion of fructose-6-phosphate (F6P) to fructose-1,6-bisphosphate (F1,6BP) [7]. PFK-1 exists in three distinct isoforms: a platelet isoform (PFKP), a liver isoform (PFKL), and a muscle isoform (PFKM). PFKP is the predominant isoform of PFK-1 in various types of tumors [8–10]. Upregulation of PFKP is strongly associated with tumor progression and is a significant indicator of an unfavorable prognosis in breast cancer, glioblastoma, and leukemia [8, 10, 11]. A substantial body of research has demonstrated the critical role of PFKP in promoting aerobic glycolysis within cancer cells, consequently driving cancer cell proliferation and metastasis [12, 13]. In addition to promoting glycolysis, emerging evidence also suggests that PFKP may influence tumor progression through other mechanisms [14]. However, the functions and regulatory mechanisms of PFKP in HNSCC remain to be fully elucidated.

c-Myc is a transcription factor involved in a wide array of biological processes, spanning cell proliferation, differentiation, epithelial-mesenchymal transition, and the modulation of the tumor microenvironment [15–17]. As a labile protein with a short half-life, c-Myc is primarily degraded through the ubiquitin-proteasome pathway [18]. The degradation of c-Myc involves a cascade of phosphorylation events, with serine 62 (S62) phosphorylated by ERK and threonine 58 (T58) regulated by GSK3β [19, 20]. Additionally, other molecules, such as ubiquitin-specific peptidases 5 and 37, also participate in the degradation process of c-Myc [21, 22]. It has been reported that c-Myc is overexpressed in HNSCC and is associated with a poor prognosis [23]. However, the mechanisms underlying the increase in c-Myc in HNSCC remain largely unknown.

Therefore, this current study aims to assess and validate the expression patterns of PFKP and c-Myc in HNSCC tumors versus adjacent normal tissues, to explore their impact on the clinical features of HNSCC, and to identify the functional roles and the underlying mechanism of a positive feedback loop between PFKP and c-Myc in progression of HNSCC. Our findings suggest that PFKP contributes to HNSCC progression by enhancing ERK-mediated c-Myc stability. Furthermore, the accumulation of c-Myc promotes the transcription of PFKP, forming a positive feedback loop that intensifies HNSCC proliferation, angiogenesis, and metastasis and may function as a potential therapeutic target of HNSCC.

## Materials and methods

### Clinical specimens

A total of 120 paired samples, including HNSCC tumor and their matched adjacent normal tissues, were collected from patients undergoing routine surgeries at the Frist Affiliated Hospital of Anhui Medical University between 2014 and 2022. The term ‘Adjacent normal tissues’ is defined as tissues that are histologically normal and located adjacent to the tumor but outside the visible abnormalities [24]. The population characteristics are provided in Supplementary Table 1. Clinical information for the donors of patient-derived xenograft (PDX) and Patient-derived organoid (PDO) models has been summarized in Supplementary Table 2. Tumor staging was determined according to the TNM classification system. All samples were obtained with the participants’ informed consent. The use of human HNSCC tissues and this informed consent has been approved by the ethics board of hospital.

### Cell line and cell culture

The HNSCC cell lines TU212 and human normal oral keratinocyte (NOK) were obtained from Otwo Biotech (Shenzhen, China). The human umbilical vein endothelial cells (HUVECs), HNSCC cell line CAL27 and HEK-293T cells were purchased from the American Type Culture Collection (ATCC, VA, USA). The human HNSCC cell lines TU177 and LIU-LSC-1 have been described previously [25, 26]. HNSCC cell lines and NOK cells were cultured in RPMI 1640 medium (Gibco, CA, USA) containing 10% fetal bovine serum (FBS, Gibco), which also contained 100 U/mL of penicillin and 100 µg/mL of streptomycin (Beyotime, Jiangsu, China). Other cells were cultured in Dulbecco’s Modified Eagle’s Medium (DMEM) (Gibco) with the same composition at 37 °C in a humidified incubator containing 5% CO_2_.

### Reagents and antibodies

MG132, Cycloheximide (CHX), EGF protein, 10,058-F4, and gefitinib were obtained from MedChemExpress (MCE, NJ, USA). Puromycin were from Sigma-Aldrich (MO, USA). All information regarding antibodies used in this study is provided in Supplementary Table 3.

### Plasmids transfection

The full-length PFKP plasmid (amino acids 1-784), truncated PFKP plasmids including PFKP-fragment 1 (amino acids 1-399) and PFKP-fragment 2 (amino acids 412–784), as well as mutant PFKP plasmids were designed and synthesized by GenePharma (Shanghai, China). The c-Myc plasmids and ERK2-HA plasmids were obtained from Tsingke Biotech (Beijing, China). The above plasmids were transfected with lipofectamine 3000 reagents (Thermo Fisher Scientific, MA, USA) according to the manufacturer’s protocol.

### RNA extraction, quantitative realtime RT–PCR (qRT-PCR) and RNA sequencing

Total RNA extraction, cDNA synthesis, qRT-PCR and RNA sequencing were performed as described previously [25]. Total RNA was isolated using TRIzol reagent (Invitrogen, CA, USA) in accordance with the manufacturer’s instructions. The quality and concentration of the extracted RNA were evaluated using a NanoDrop 2000 spectrophotometer (Thermo Fisher Scientific). Subsequently, 1 µg of total RNA was converted into first-strand cDNA using a RevertAid™ First Stand cDNA Synthesis Kit (Fermentas, MA, USA). Quantitative real-time PCR (RT-PCR) was conducted using SYBR Premix Ex TaqTM II (TaKaRa, Kyoto, Japan) on a LightCycler^®^ 96 system (Roche, Switzerland), following the manufacturer’s protocol. The expression levels of the target genes were normalized to β-actin using the 2^−ΔΔCt^ formula. The primers for qRT-PCR (provided by Sangon Biotech, Shanghai, China) are shown in Supplementary Table 4.

The total RNA from shPFKP LIU-LSC-1 and their control cells were isolated using TRIzol Reagent, followed by quality control using NanoDrop1000 and Bioanalyzer 2100 (Agilent, CA, USA). Poly (A) mRNA was captured using Oligo(dT) beads (Thermo Fisher), fragmented, and reverse transcribed into cDNA. The cDNA was then synthesized into double-stranded DNA, end-repaired, A-tailed, ligated with adapters, and size-selected using magnetic beads. After UDG treatment and PCR amplification, the final cDNA library was sequenced on an Illumina NovaSeq™ 6000 (LC Sciences, Hangzhou, China) following the vendor’s recommended protocol. The raw data in fastq format underwent quality control using fastp software. HISAT2 was used to align the sequencing data to the Homo sapiens GRCh38 reference genome, resulting in BAM files. String Tie was employed for transcript assembly and quantification using FPKM. Differential gene analysis between samples was conducted using the R package edgeR, defining differential expressed genes as those with fold change > 2 or fold change < 0.5 and a p value < 0.05. The list of differentially expressed genes (DEGs) was provided in Supplementary Table 5. Xiantao online tools was used to conduct Kyoto Encyclopedia of Genes and Genomes (KEGG) pathway and Gene Ontology (GO) enrichment analysis (https://www.xiantaozi.com/). The KEGG pathways and the gene associated with each pathway was demonstrated in Supplementary Table 6. The GO enrichment analysis was shown in Supplementary Table 7. The original data are available at NCBI Gene Expression Omnibus (GEO) under accession number GSE248242.

### Lentivirus infection

All of the lentiviral vectors were provided by GenePharma (shanghai, China), including LV4 lentiviral plasmid expressing PFKP cDNA, c-Myc cDNA and the empty plasmid; LV-2 N lentiviral shRNA expression vector targeting PFKP, c-Myc and the control scrambled shRNA (shSc). The detailed information of the target sequences was listed in Supplementary Table 8. Lentivirus production and the generation of stable cell lines were described previously [26].

### RNA interference

All siRNAs were designed and synthesized by GenePharma. Cells were transfected with siRNAs at a confluence of 50–60% using Lipofectamine RNAiMax (Thermo Fisher Scientific) according to the manufacturer’s instructions. The target sequences used were: siNC, 5’-UUCUCCGAACGUGUCACGUTT-3’; siPFKP, 5’-GGCUGAAGGAGCAAUUGAUTT-3’; siERK2, 5’- GCUGCAUUCUGGCAGAAAUTT-3’.

### Western blotting

Western blotting analysis was conducted as previously described [27]. In brief, cells and tissues were denatured and subjected to electrophoresis using a 10% SDS-polyacrylamide gel (SDS-PAGE), followed by transfer onto PVDF membranes (Millipore, Billerica, MA, USA). After blocking with 5% skimmed milk for 1 h, the membranes were then incubated with primary antibodies overnight at 4 °C. Subsequently, the blots were incubated with secondary antibodies at room temperature for 1 h and visualized using chemiluminescence.

### PFK-1 activity assay

The Phosphofructokinase (PFK) Activity Assay kit (Abcam, Cambridge, UK) was employed as per the manufacturer’s guidelines. Initially, cells grown to 80% confluence were washed with PBS and lysed using 200 µL of ice-cold PFK Assay buffer. Following homogenization and centrifugation, the supernatant was collected and the volume adjusted to 50 µL for further analysis. This prepared sample was then transferred to a 96-well plate, to which 50 µL of reaction mix was added. The assay plate was incubated at 37 °C and the absorbance at 450 nm was measured at 5-minute intervals using a microplate reader to monitor the reaction progress. PFK activity was quantified based on the NADH production, as measured against a standard curve.

### Glucose uptake assay, lactate assay

The Glucose Uptake Assay Kit (Abcam) was utilized following the manufacturer’s protocol. Initially, the cells were starved overnight in serum-free medium to enhance glucose uptake. This was followed by 20-min incubation in Krebs- Ringer-Phosphate-Hepes buffer (Thermo Fisher Scientific) containing 2% bovine serum albumin. Subsequently, 2-deoxy-d-glucose (2-DG) was added to the medium for 20 min. The cells were lysed using the extraction buffer, frozen at -80 °C for 15 min, and then heated at 85 °C for 40 min. After cooling, the neutralization buffer was added. The lysates were centrifuged at 12,000 rpm for 5 min, and the supernatant was collected. Glucose uptake was quantified by the measurement of the absorbance at 412 nm using a microplate reader.

For the lactate assay (Abcam). 2 × 10^6^ cells per assay were processed in ice-cold lactate assay buffer, centrifuged to collect the supernatant, and deproteinized using the TCA kit (Abcam). After adding the reaction mix, the samples incubated at room temperature for 30 min and lactate levels were then measured at OD 450 nm using a microplate reader.

### Cell viability assays

The HNSCC cells were seed in 96-well plates at a density of 1.5 × 10^3^ cells/well and cell viability was measured by cell counting kit-8 (CCK-8, Topscience, shanghai, China) at 24, 48, 72, and 96 h, respectively. The optical density (OD) values were measured at 450 nm after incubation with CCK-8 reagent at 37℃ for 2 h.

### Colony formation assay

Cells were seeded in triplicate in 6 cm dishes (1000 cells/dish) and cultured for 2 weeks. cells were fixed with 4% polyformaldehyde, stained with 0.5% crystal violet (Beyotime), and photographed with a digital scanner after drying.

### Cell cycle and apoptosis analysis

Cell cycle and apoptosis assays were measured by the cell cycle analysis kit (beyotime) and Annexin V-APC/PI apoptosis detection kit (Keygen, Jiangsu, China) according to the manufacturer’s protocols. The cells were analyzed with a flow cytometer (Beckman Coulter, CA, USA). Data was analyzed using the Flowjo software v10.

### HPV detection

Genomic DNA was extracted from frozen tissue samples using the Animal Genomic DNA Kit (Tsingke Biotech), following the manufacturer’s instructions. The PCR assays were conducted using the GP5+/6 + and MY09/11 primers, as previously described [28]. Amplification comprised an initial denaturation step at 95 °C for 5 min, followed by 40 cycles at 95 °C for 1 min, annealing at 40 °C for 2 min, and chain elongation at 72 °C for 1 min. The final cycle included a 4 min elongation step at 72 °C. Subsequently, the resulting fragments were analyzed via agarose gel electrophoresis. The primer sequences utilized were listed in Supplementary Table 4.

### Cell migration and invasion assays in vitro

Transwell assays were divided into transwell migration and transwell invasion assay, 40 µL Matrigel (Corning, NY, USA) was used to coat the upper membrane in advanced for transwell invasion assay. 200 µL serum-free cell suspension (2 × 10^4^ cells per insert) was seeded in the upper chamber, and the medium with 20% FBS was supplemented to the lower chamber. After incubation at 37℃ for 24 h, the cells were fixed with 4% paraformaldehyde, and stained with 0.1% crystal violet, then cells migrated to the lower side of the filter were imaged and counted.

### Tube formation assay

Cell-derived conditional medium (CM) was prepared as described previously [26]. Briefly, stably transfected cells were seeded in a 6 cm dish and incubated for 48 h, after which the medium was replaced by a fresh serum-deprived medium. After 24 h of incubation, the medium was collected, filtered, and then concentrated by ultrafiltration. The CM was stored at -80℃ until used.

For the tube formation assay, a total of 150 µL Matrigel (Corning) was added to a 48-well plate and incubated at 37℃ for 30 min. Then, HUVECs (3.5 × 10^4^) in 300 µL of prepared medium were added to each well and incubated at 37 °C in 5% CO_2_. After incubation for 12 h, bright-field images were recorded using a microscope and analyzed with WimTube (https://www.wimasis.com/en/WimTube, Wimasis GmbH, Munich, Germany).

### Dual-luciferase reporter assay

A DNA fragment containing the promoter of the PFKP-encoding gene was inserted into pGL3 Basic Vector (Promega, WI, USA). The potential c-Myc binding sites on the promoter of the PFKP gene were mutated using the Q5 site-directed mutagenesis kit (NEB, MA, USA). All used primers were listed in Supplementary Table 9. HEK-293T cells were seeded in 24-well plates and co-transfected the wild-type or the mutated promoter constructs (200 ng) and pRL-TK luciferase (10 ng) using FuGENE (Promega). Then, c-Myc-pcDNA 3.0 or empty vector pcDNA 3.0 was transfected into cells using Lipofectamine 3000 (Thermo Fisher Scientific). The luciferase activity was estimated using the Dual-Luciferase Reporter Assay System (Promega).

### Chromatin immunoprecipitation (ChIP)

ChIP assay was performed using a simpleChIP^®^ Plus Enzymatic Chromain IP kit (Cell signaling Technology, MA, USA) as described previously [25]. In brief, cells were crosslinked by 1% formaldehyde and chromatin was extracted and sheared by Ultrasonic crusher (Thermo Fisher Scientific). Samples were immunoprecipitated with anti-c-Myc antibody 4℃ overnight. The immunoprecipitated DNA was purified and analyzed by PCR or qRT-PCR with primers. The primer sequences were listed in Supplementary Table 10.

### In vivo xenograft assay

All animal experiments were performed according to protocols approved by the Experimental Animal Ethical Committee of Anhui Medical University. BALB/c nude mice and NOD/SCID mice were purchased from GemPharmatech (Jiangsu, China).

For the subcutaneous tumor assay, 4 × 10^6^ PFKP-knockdown LIU-LSC-1 cells, PFKP-overexpressing TU177 cells, and their corresponding control cells were suspended in 0.2 ml PBS and injected into the flanks of BALB/c mice. Tumor growth was examined every 3 days. Tumor volume was calculated using the following equation: Volume = (length×width^2^)/2 (mm^3^). Mice were sacrificed after 4 weeks, and the tumor tissues were separated, weighed, and photographed, followed by immunohistochemical (IHC) staining of the tissues.

LIU-LSC-1 cell-derived xenograft (CDX) models and HNSCC patient-derived xenograft (PDX) models were used to test the synergistic therapeutic effect of inhibiting PFKP and c-Myc in HNSCC. For CDX models, cultured LIU-LSC-1 cells (4 × 10^6^) were subcutaneously injected into the right flank of all mice. When the tumor volume was 100 mm^3^, the mice were randomly divided into four groups (*n* = 5 per group) and treated with PFKP siRNA (100 µg, twice/week) or control siRNA (siNC), together with 10,058-F4 (3 mg/kg, every 3 days) or vehicle. 10,058-F4 was dissolved in vehicle (10% DMSO, 40% PEG300, 5% Tween80, and 45% saline) and intraperitoneally injected. The siRNAs (GenePhama, Jiangsu, China) were injected directly into the tumor at two or more spots each time. For HNSCC PDX models, an HNSCC tissue sample with high expression of PFKP and c-Myc was selected. Establishment of HNSCC PDX models has been described previously [25]. When the third-passage (P3) tumor size reached approximately 100 mm^3^, the mice were randomly divided into four groups. The subsequent procedures were the same as for the CDX model.

### Lung metastasis mouse model

For the tail vein injection pulmonary metastasis model, NOD/SCID mice (GemPharma) were randomly divided into groups (5 mice per group). 1 × 10^6^ genetically engineered cells were suspended in 0.1 ml PBS and injected into the lateral tail vein. Mice were killed after 7 weeks, and lungs were extracted and fixed in 4% paraformaldehyde. Visible lung metastases were assessed and counted. Lung tissue was embedded in paraffin, sectioned and stained with hematoxylin and eosin (H&E).

### Chicken Chorioallantoic membrane (CAM) assay

CAM assay was performed as previously described [29]. Briefly, Sterile gelatin sponges mixed with 20 µL of suspension containing 2 × 10^6^ HNSCC cells were planted on CAM. After a week, the CAM was separated from the eggs after fixation with stationary solution (methanal: acetone, 1:1) for 30 min. Then, the CAM was recorded by a digital camera.

### H&E, IHC and immunofluorescence (IF) assay

H&E and IHC staining was performed as previously described [25]. Tissues or organoids were fixed in 4% paraformaldehyde overnight, dehydrated, and embedded in paraffin. Sections were subjected to H&E as well as IHC staining. Antibodies against PFKP (diluted 1:50), c-Myc (diluted 1:100), Ki-67 (diluted 1:100) and CD31 (diluted 1:100) were used. The modified histologic score was used to evaluate the IHC staining of PFKP, c-Myc, and CD31, respectively. The H-scores was calculated as [{% of weak staining} × 1] + [{% of moderate staining} × 2] + [{% of strong staining} × 3]), resulting in a score between 0 and 300 for each staining. For the evaluation of Ki-67 IHC staining, the staining intensity was measured by quantifying the percentage of cells with nuclear staining using Image J software.

For IF assays, cells were fixed with 4% formaldehyde, treated with 0.5% Triton X-100 (Sigma-Aldrich) for permeabilization, and then blocked with Immunol Staining Blocking Buffer (Beyotime). Cells were incubated in a primary antibody overnight and followed with fluorochrome-conjugated secondary antibodies (Cell Signaling Technology, MA, USA) for 1 h. Before imaging, dishes were mounted using ProLong Gold Antifade Reagent with DAPI (Thermo Fisher Scientific).

### Establishment and culture of HNSCC organoids

HNSCC organoid cultures were established and maintained as described previously with minor modifications [30, 31]. Briefly, fresh tumor tissues obtained from HNSCC patients were washed in ice-cold PBS three times for 5 min and then cut into 1-3mm^3^ fragments on ice. The tissue fragments were enzymatically digested using trypsin (Sigma-Aldrich) for approximately 30 min. Once the mixture became cloudy, it was passed through a 100-µm cell strainer and centrifuged at 200×g for 5 min to collect the cell clusters, which were then washed with PBS three times for 5 min to remove residual digestive enzymes. After the final wash, the cell clusters were embedded in Matrigel (Corning). Following solidification, the Matrigel-cell mixture was overlaid with HNSCC organoid medium (BioGenous, Jiangsu, China) and incubated at 37 °C in a CO_2_ incubator. The medium was refreshed every 3–5 days.

For lentiviral infection, the organoids were dissociated into cell clusters using a pipette. After centrifugation at 200×g (4 °C) for 5 min, the cell clusters were suspended in media (BioGenous) containing either control or PFKP-knockdown lentivirus particles. They were then spin-infected in centrifuge tubes (700×g at 25 °C for 90 min) and then incubated at 37 °C for 4 h. Finally, the mixture was centrifuged at 300×g for 5 min, and the cell clusters were embedded in Matrigel. The efficiency of PFKP knockdown was verified by Western blotting.

For drug tests, HNSCC organoids in good condition were inoculated in a 96-well cell culture plate at an appropriate density and covered with 100 µL of culture medium. After overnight incubation, the culture medium was replaced with the indicated concentrations of DMSO or 10,058-F4. Each group contained three multiple wells. After 4 days of treatment, images of organoids were captured, and their diameters were assessed using image analysis software (PhotoShop, CA, USA).

### Mass spectrometry

The cell lysate was centrifuged to collect the sample supernatant. Meanwhile, anti-PFKP or IgG antibodies were incubated with agarose beads at room temperature for 1 h. Subsequently, the processed sample supernatant was added to the agarose beads and left overnight at 4 °C. After washing, the samples were separated on a 4–12% SDS-PAGE gel. The gel bands were cut out and collected. The samples were sent to Bioprofile (Shanghai, China) for liquid chromatography-mass spectrometry (LC/MS) analysis to identify proteins that interact with PFKP.

#### Co-immunoprecipitation

Co-immunoprecipitation (CoIP) was performed with a commercial Kit (Beyotime) following the manufacturer’s protocol. Cells were lysed in buffers with protease inhibitor, incubated on ice for 30 min, and centrifuged at 4 °C for 5 min. Cell lysates were incubated overnight at 4 °C with protein G agarose gel and the indicated primary antibody or control IgG. The agarose gels were washed three times in lysis buffer and then boiled for 5 min at 95 °C. Subsequently, protein co-precipitation was analyzed using Western blotting or LC-MS (Thermo Fisher Scientific). All antibodies used in the present study are listed in Supplementary Table 3.

### Molecular docking

The crystal structures of the domain 2 fragment of PFKP and ERK2 were obtained from the RCSB PDB protein data bank (http://www.rcsb.org/pdb/). The acquired protein crystals underwent processing using Schrödinger software’s Protein Preparation Wizard module, involving various treatments such as protein pre-processing and removal of water molecules. The protein–protein docking module was utilized to analyze docking between the processed domain 2 fragment of PFKP and ERK2. The ligand rotations were set to 70,000, and the maximum number of returned poses was 30. A lower free energy of binding between the ligand and the receptor indicated higher binding stability.

### Ubiquitination assay

Cells in a logarithmic growth phase were seeded in 10 cm dishes and cultured for 24 h. Then, the cells were treated with 10 µM MG-132 for 8 h. Subsequently these cells were washed with PBS and lysed for immunoprecipitation assay using anti-c-Myc antibody followed by Western blotting assay using anti-Ub antibody to detect c-Myc ubiquitination.

### Bioinformatics analysis

HNSCC mRNA data and corresponding clinical data were downloaded from the TCGA database (https://portal.gdc.cancer.gov/). We adopted the log-rank test and Kaplan–Meier (K-M) survival analysis to analyze survival distribution. Gene Set Enrichment Analysis (GSEA) is used to explore the MYC-related signaling pathways associated with PFKP. In the TCGA-HNSCC cohort, the patients were divided into high- and low-expression groups based on the median of PFKP values. Differentially expressed genes between the high and low groups were analyzed using the ‘limma’ package, and all genes were ranked according to log_2_FC. GSEA was conducted using this ranked gene list against the HALLMARK_MYC_TARGETS_V1 and HALLMARK_MYC_TARGETS_V2 gene sets from the Molecular Signatures Database (Broad Institute, Cambridge, MA) through the ‘clusterProfiler’ package. False discovery rate (q-values) < 0.25 were considered statistically significant [32].

### Statistical analysis

Statistical analyses were conducted in GraphPad Prism 8.0 (CA, USA). Statistical significance was calculated using paired/unpaired Student’s *t*-tests or One-way ANOVA as appropriate. Pearson’s correlation analysis was used to assess the correlation between the two sets of data. Survival rates were calculated with the K-M method. Each experiment was performed at least three times. Statistical significance was set at *P* < 0.05.

## Results

### PFKP is highly expressed in tumors and predicts poor survival of HNSCC patients

**Fig. 1:**
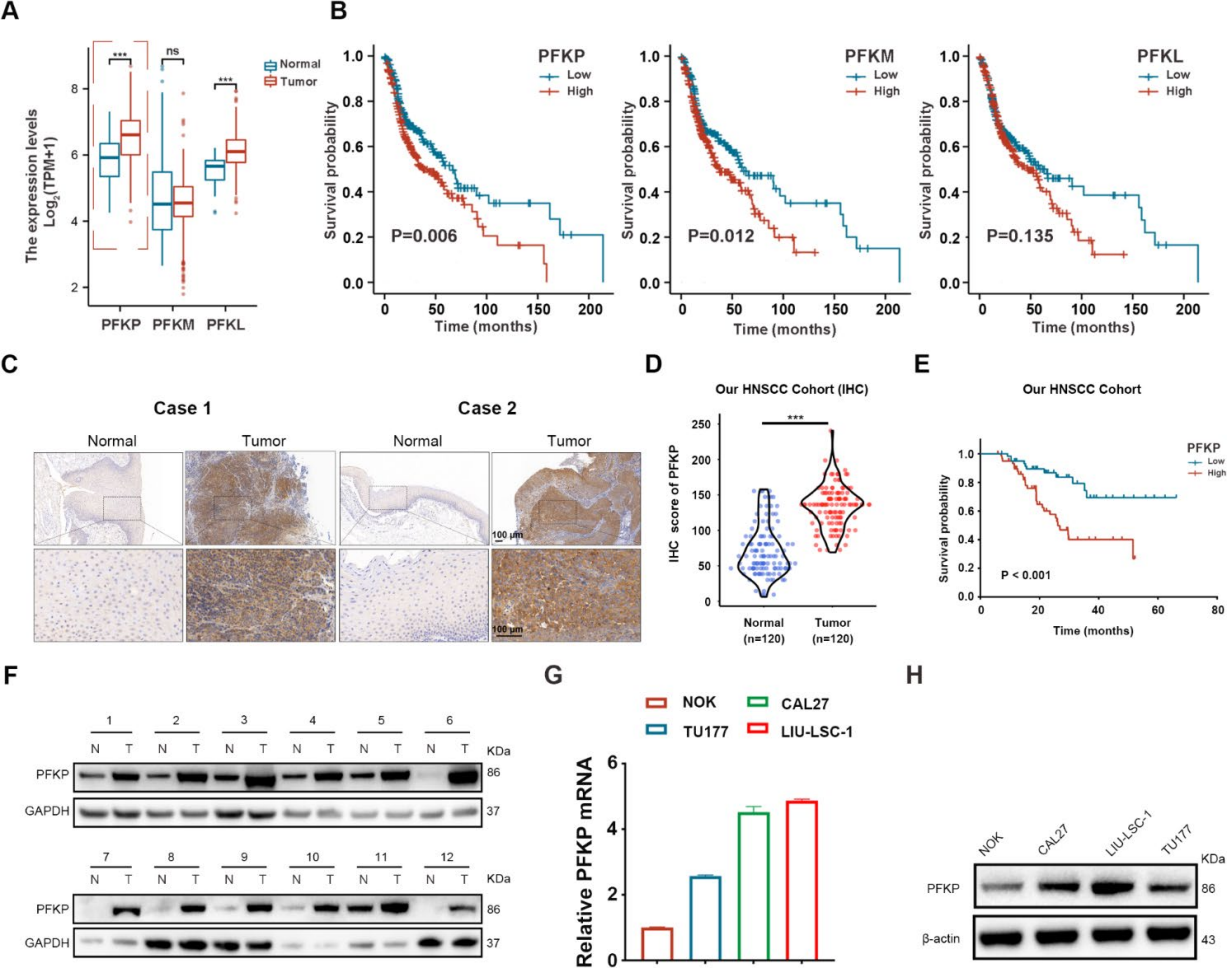
PFKP expression is upregulated in HNSCC and associated with poor prognosis. (**A**) Expression analysis of PFKP, PFKM, and PFKL in tumor and normal tissues using TCGA-HNSCC. (**B**) K-M plot for OS of HNSCC patients in TCGA dataset based on PFKP, PFKM and PFKL expression. High (*n* = 251) and low (*n* = 250) PFKP, PFKM and PFKL expression were stratified by the median expression level. (**C**) Representative IHC images illustrating PFKP staining in normal and tumor tissues. Scale bar, 100 μm. (**D**) Statistical analysis of the IHC results. (**E**) K-M plot depicting OS in HNSCC patients from our cohort. (F) Western blotting analysis of PFKP protein levels in paired normal and tumor tissues (*n* = 12). (**G**) and (**H**) Gene and protein expression of PFKP in NOK, CAL27, TU177, and LIU-LSC-1 cells, as detected by the qRT-PCR (**G**) and Western blotting (**H**). The error bars represent the mean ± SD of triplicate technical replicates. ***P < 0.001

We utilized the TCGA-HNSCC database to analyze the expression profiles of the three PFK-1 isoforms (PFKP, PFKM, and PFKL) and their association with patient survival. Notably, only PFKP exhibited elevated expression levels in HNSCC tissues, and this heightened expression was indicative of poor prognosis in HNSCC patients (Fig. 1A and B). To explore the significance of PFKP in HNSCC, we initially assessed PFKP levels in 120 HNSCC tissues sample and their corresponding adjacent normal tissues using IHC staining (Fig. 1C). Remarkably, the tumor tissues exhibited elevated PFKP expression levels (Fig. 1D). Representative IHC images for the different PFKP levels in HNSCC tissues were provided in Supplementary Fig. 1. Our HNSCC cohort data revealed a statistically significant association between increased PFKP expression and poor OS of HNSCC patients (Fig. 1E). To provide a more comprehensive depiction of PFKP protein levels, we chose 12 pairs samples from the patient cohort for Western blotting analysis, confirming the high expression of PFKP in HNSCC samples (Fig. 1F). We also examined the PFKP mRNA and protein levels in three HNSCC cell lines and NOK cell lines using the qRT-PCR and Western blotting. Compared with NOK cells, the HNSCC cell lines showed higher levels of PFKP expression (Fig. 1G and H). Taken together, these data indicate that PFKP is upregulated in HNSCC tissues and cell lines and high PFKP expression predicts a poor prognosis for patients with HNSCC.

### PFKP enhances tumorigenic capacity of HNSCC cells in vitro

**Fig. 2:**
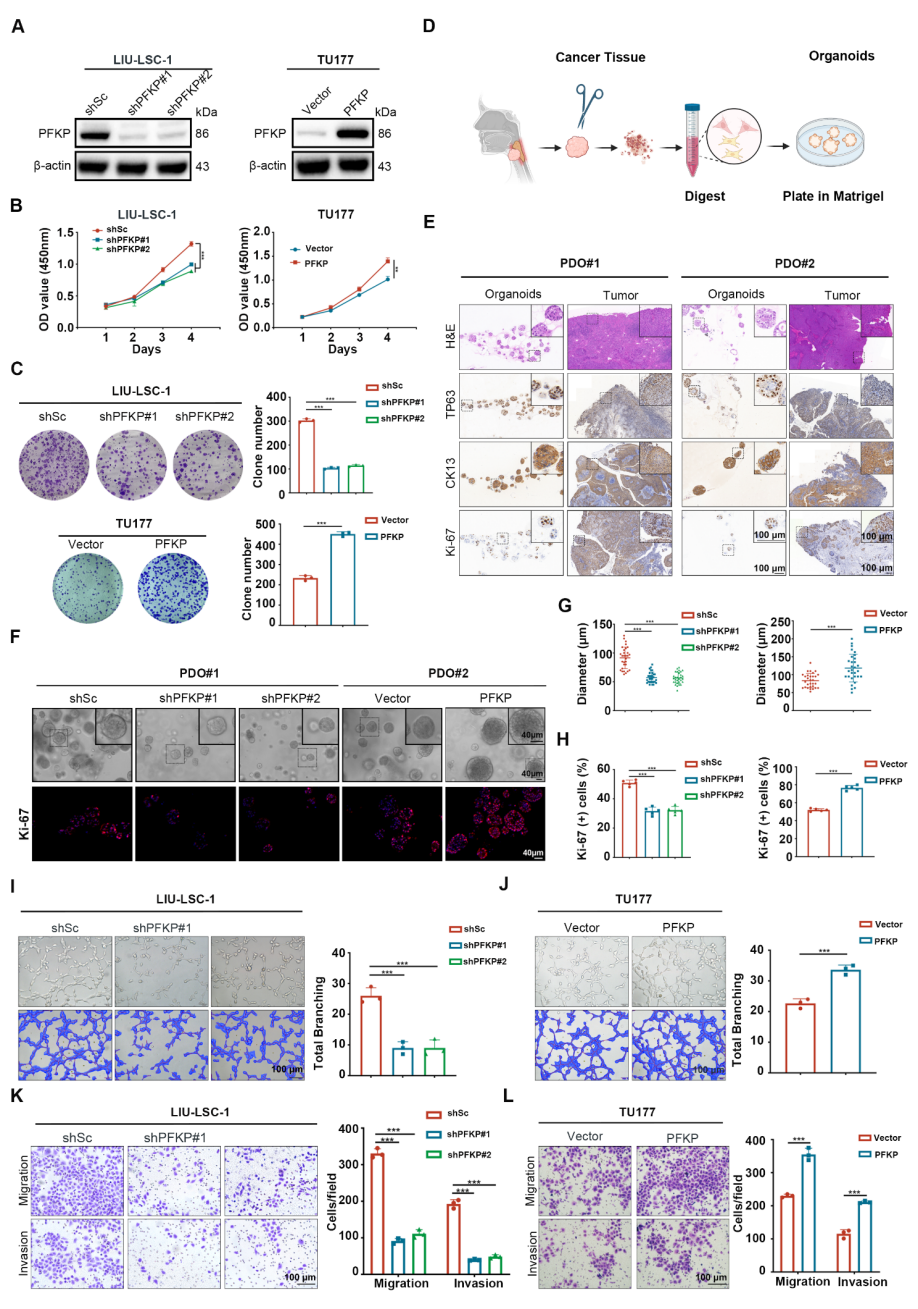
PFKP promotes the proliferation, angiogenesis, migration, and invasion of HNSCC cells. (**A**) LIU-LSC-1 cells were transduced with a lentivirus expressing shRNAs against PFKP (shPFKP#1 and shPFKP#2) or a scrambled sequence (shSc). TU177 cells were infected with control (vector) lentiviruses or lentiviruses encoding PFKP. The expression of PFKP was assessed by Western blotting. (**B**) CCK-8 assays were performed to evaluate cell growth rates of the indicated cells. (**C**) The effects of PFKP inhibition and overexpression on colony-forming ability were detected by colony-formation assays. (**D**) Schematic outline of the digestion and initial culture conditions of HNSCC organoids. (**E**) H&E staining and immunostaining for CK13, TP63, and Ki-67 of organoids and control tissue. Scale bars, 100 μm. (**F**) Representative images of organoid diameter and Ki-67 immunofluorescence intensity after knockdown or overexpression of PFKP. Scale bar, 40 μm. (**G**) Statistics of tumor organoid diameter (*n* = 30). (**H**) Bar graph of the proportion of Ki-67 + cells per organoid (*n* = 5). (**I**-**J**) The effect of PFKP on angiogenesis was determined by a tube formation assay. Representative image (left panels) and quantifications (right panels). Scale bar, 100 μm. (**K**-**L**) Transwell assays showed the migration and invasion rates in PFKP-knockdown LIU-LSC-1 cells and PFKP-overexpressing TU177 cells. Scale bar, 100 μm. Error bars indicate mean ± SD of triplicate (unless mentioned otherwise) samples. **P < 0.01; ***P < 0.001

To investigate the function of PFKP, we performed knockdown experiments in LIU-LSC-1 cells, in which PFKP is highly expressed, and ectopically expressed PFKP in TU177 cells, in which PFKP is lowly expressed. The efficiency of knockdown and overexpression was confirmed by Western blotting (Fig. 2A). Subsequently, as depicted in Fig. 2B and C, both short-term proliferation assays and long-term clonogenic assays indicated a decrease in the proliferation in LIU-LSC-1 cells following PFKP knockdown, and increased cell proliferation in TU177 when PFKP was overexpressed. Furthermore, we established two independent HNSCC PDOs to validate the oncogenic role of PFKP (Fig. 2D). As shown in Fig. 2E and Supplementary Fig. 2A, HNSCC organoids (HPV negative) faithfully recapitulated the characteristics of primary tissues. IHC staining was conducted to assess PFKP expression in two cases of HNSCC tissue, and Western blotting was used to measure PFKP levels in tissues and PDOs (Supplementary Fig. 2B and 2 C). Organoids with a high level of PFKP (PDO#1) exhibited decreased growth following PFKP depletion (Supplementary Fig. 2D and Fig. 2F and 2G). Conversely, for organoids characterized by a low level of PFKP (PDO#2), overexpression of PFKP markedly increased their growth (Supplementary Fig. 2D and Fig. 2F and 2G). Ki-67 staining analysis revealed that the proportion of Ki-67-positive cells increased in line with the level of PFKP protein expression in each organoid (Fig. 2F and H).

Given that PFKP is an isoform of PFK-1, we examined the impact of PFKP on the activity of PFK-1 and glycolysis. As shown in Supplementary Fig. 3A-C, depletion of PFKP weakened the activity of PFK-1 and decreased glucose uptake and lactate production in LIU-LSC-1 cells. Furthermore, we observed that PFKP suppression led to cell cycle arrest, with minimal impact on apoptosis in LIU-LSC-1 cells. (Supplementary Fig. 3D and 3E).

Since PFKP is associated with tumor angiogenesis in cancers [33, 34], we explored its role in the vascular formation capacity of HNSCC cells. An in vitro capillary tube formation assay was conducted, and the results revealed that HUVECs cultured with CM derived from PFKP-knockdown cells exhibited reduced formation of capillary-like structures and branch points compared to the control cells (Fig. 2I). In contrast, CM obtained from TU177 cells with ectopic PFKP expression had the opposite effects (Fig. 2J). Furthermore, we confirmed that VEGFA, the key regulator of angiogenesis, is positively regulated by PFKP (Supplementary Fig. 3F). These findings together indicate that PFKP may play a pro-angiogenic role in HNSCC.

Additionally, to determine the roles of PFKP in the migration and invasion of HNSCC cells, we conducted in vitro migration and invasion assays. We found that PFKP suppression significantly inhibited the migration and invasion of LIU-LSC-1 cells as shown in Fig. 2K. In contrast, the ectopic expression of PFKP in TU177 cells enhanced their migration and invasion abilities (Fig. 2L). Given the role of epithelial-mesenchymal transition (EMT) in migration and invasion [35], we analyzed EMT markers (N-cadherin, vimentin, and snail) in cells with PFKP knockdown or overexpression. Our results indicated that PFKP knockdown reduced the expression of N-cadherin, vimentin, and snail, respectively, whereas PFKP overexpression produced contrary effects (Supplementary Fig. 3G). Thus, PFKP may enhance the cell proliferation, migration, invasion, angiogenic capabilities and glycolysis of HNSCC cells in *vitro*.

### PFKP promotes HNSCC tumor progression in vivo

**Fig. 3:**
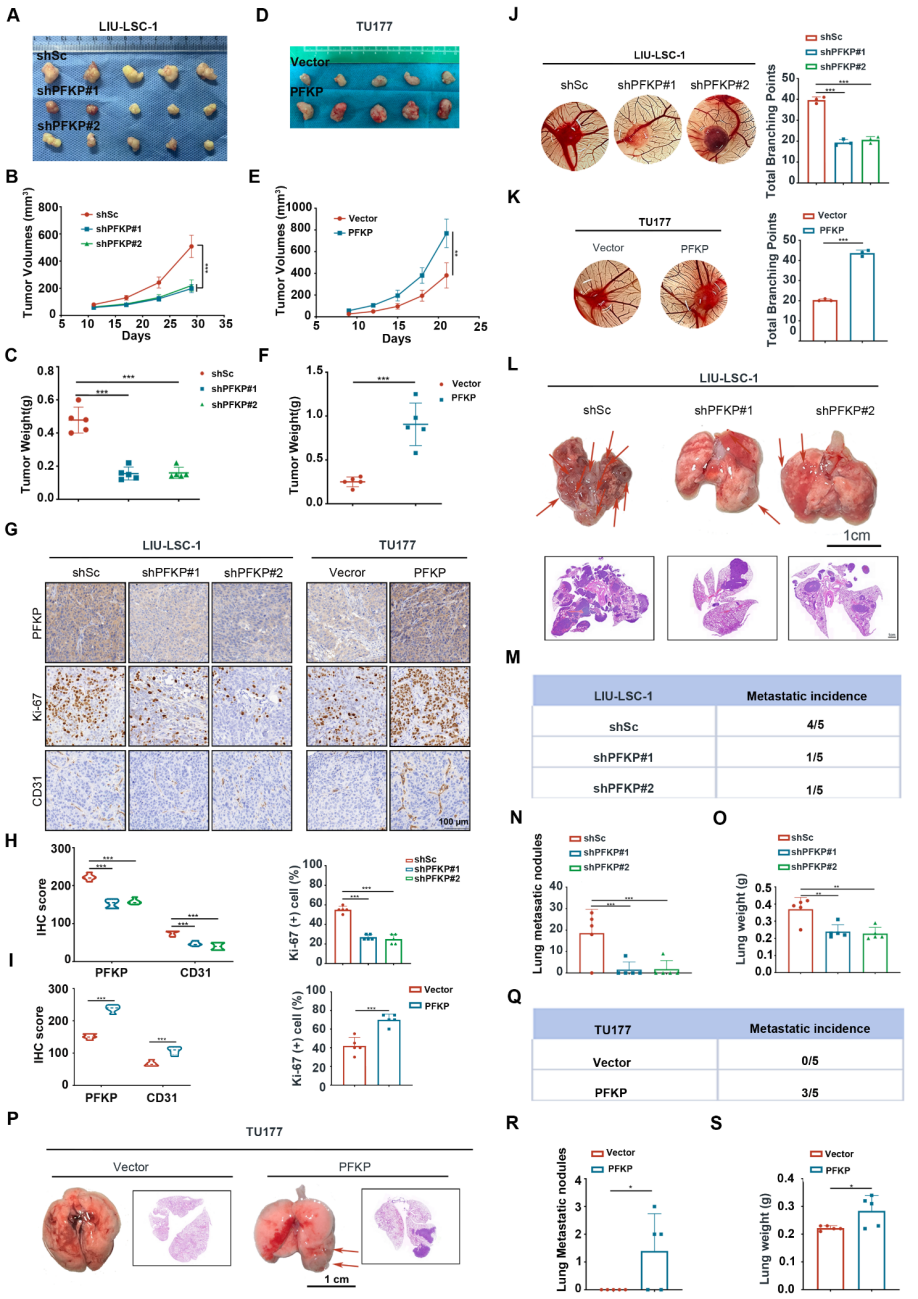
The tumor-promoting effects of PFKP in vivo. (**A**-**G**) Subcutaneous inoculation of designated cells into nude mice was monitored for tumor growth. (**A**, **D**) Tumor images, (**B**, **E**) tumor volumes, (**C**, **F**) tumor weight, and (**G**) representative IHC images for PFKP, Ki-67, and CD31 in xenograft tissues. Scale bars, 100 μm. (H-I) the quantitative analysis results for PFKP, CD31 and Ki-67. Error bars indicate mean ± SD. ***P < 0.001. (**J**-**K**) Angiogenic effects of PFKP assessed via a CAM assay: Representative images (left panels) and statistical analysis (right panels). Error bars represent mean ± SD (*n* = 3 per group). ***P < 0.001. (**L**) The indicated LIU-LSC-1 cells were injected into nude mice. The figure shows lung images and H&E-stained sections from PFKP knockdown and control cell models. (**M**-**O**) Lung metastasis data: (**M**) Number of mice with metastases, (**N**) Number of lung nodules, (**O**) Lung weights. (**P**) Lung images and H&E-stained sections from a model using TU177 cells overexpressing PFKP and controls. (**Q**-**S**) Lung metastasis outcomes for PFKP overexpression: (**O**) Mice with metastases, (**R**) Lung nodules, (**S**) Lung weights. Error bars indicate mean ± SD (*n* = 5 mice/group). *P < 0.05. **P < 0.01; ***P < 0.001

To assess the *in* vivo function of PFKP, we performed xenograft assays. As shown in Fig. 3A-C, the suppression of PFKP markedly inhibited tumor growth compared to the control, as reflected by the decreased tumor volume and lower tumor weight in the PFKP-knockdown group compared to that in the control group. IHC staining showed that depletion of PFKP significantly reduced both Ki-67 (a marker of proliferation) and CD31 (an angiogenic marker) expression in tumor tissues (Fig. 3G, left panel and Fig. 3H). In contrast, PFKP overexpression promoted tumor growth in the xenograft model assay (Fig. 3D-F and G, right panel and Fig. 3I).

To confirm the pro-angiogenic role of PFKP in HNSCC in vivo, we performed a CAM assay. As shown in Fig. 3J-K, inhibition of PFKP in LIU-LSC-1 cells led to a decreased angiogenic response compared to control cells. Conversely, overexpressing PFKP in TU177 cells facilitated blood vessels formations towards the graft, in contrast with control cells.

Furthermore, we investigated PFKP’s effects on metastasis using a tail vein injection pulmonary metastasis model. We observed a significant reduction in the number of metastatic nodules in the PFKP-knockdown group compared to the control group (Fig. 3L-N). Consistently, the wet lung weight of mice bearing PFKP-knockdown LIU-LSC-1 cells was lower than that in the control group (Fig. 3O). Moreover, in contrast to the corresponding control group, the PFKP-overexpressing group exhibited increased lung metastasis (Fig. 3P-R) and had a heavier wet lung weight (Fig. 3S). These findings suggest that PFKP may promote HNSCC tumor growth, angiogenesis, and metastasis in vivo.

### PFKP interacts with ERK2 to activate the MAPK/ERK pathway

**Fig. 4:**
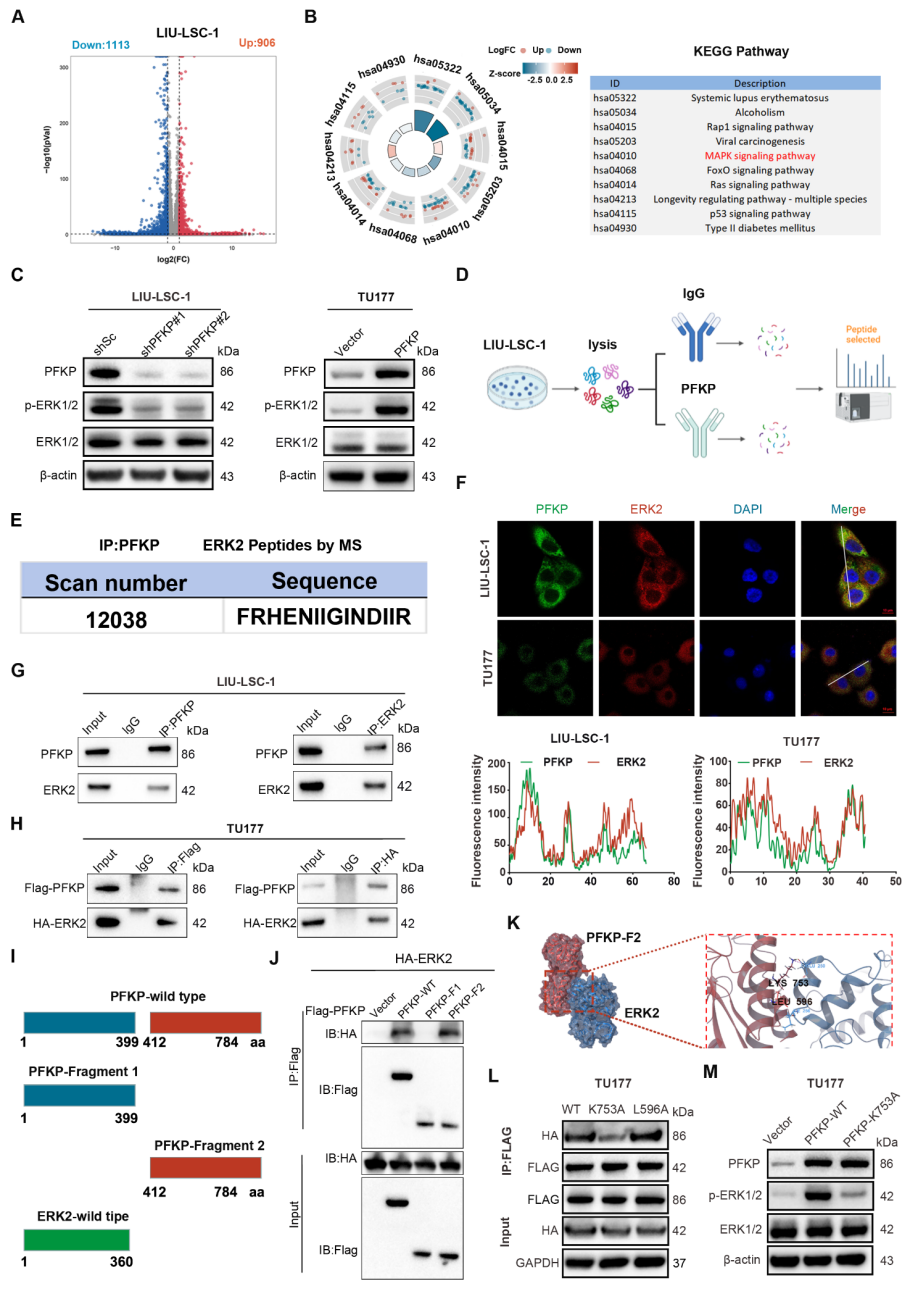
PFKP activates the MAPK/ERK pathway via ERK2. (**A**) Volcano plots showing differentially expressed genes (DEGs) expression (|log_2_FC| > 1 and *p* < 0.05) between shPFKP LIU-LSC-1 cells and the control cells. (**B**) KEGG pathway analysis showing the top 10 enriched pathways of DEGs. (**C**) Effect of PFKP depletion or overexpression on p-ERK1/2 and ERK1/2 levels in HNSCC cells by Western blotting. (**D**) Schematic workflow of the IP-PFKP experiments. (**E**) IP of PFKP, followed by MS analysis, to identify ERK2 binding to PFKP. (**F**) Representative immunofluorescence images showing the co-localization of PFKP and ERK2 in HNSCC cells. The intensity profiles of PFKP and ERK2 along the white line were plotted. Scale bars, 10 μm. (**G**) Co-IP assays reveal the endogenous interaction between PRKP and ERK2 in LIU-LSC-1 cells. (**H**) TU177 cells were co-transfected with FLAG-PFKP and HA-ERK2 plasmids and subjected to IP for FLAG and HA, respectively. (**I**) Schematic structure of the PFKP full-length protein and domains. (**J**) The co-IP assay to assess the interaction between ERK2 and different domains of PFKP. (**K**) Molecular docking model of ERK2 interacting with domain 2 of PFKP. (**L**) Co-IP assay to identify the amino acid sites of PFKP binding to ERK2 in TU177 cells with different PFKP mutants. (**M**) Effect of PFKP mutation on p-ERK and ERK levels in HNSCC cells by Western blotting

To elucidate the potential mechanism through which PFKP functions in HNSCC, we conducted RNA-seq analysis using the PFKP-knockdown LIU-LSC-1 cells and control cells. Our results revealed 2019 DEGs, among which 906 were upregulated and 1113 were downregulated (Fig. 4A and Supplementary Table 5). The KEGG enrichment analysis for these DEGs identified the MAPK pathway as one of the significantly altered pathways following PFKP suppression (Fig. 4B and Supplementary Table 6). The ERK pathway is the most important signaling cascade among all MAPK signal transduction pathways and plays a crucial role in the survival and development of tumor cells [36]. Activation of the ERK cascade is pervasive across various cancer types, with phosphorylated ERK1/2 (p-ERK1/2) present in over 90% of HNSCC patients [37, 38]. The suppression of PFKP in LIU-LSC-1 cells resulted in decreased levels of p-ERK1/2, whereas PFKP overexpression in TU177 cells led to elevated p-ERK1/2 levels (Fig. 4C). Furthermore, we performed an IP of PFKP followed by MS analysis to identify proteins interacting with PFKP (Fig. 4D) (e.g., ERK2) (Fig. 4E and Supplementary Fig. 4). An IF assay indicated that PFKP may co-localize with ERK2, predominantly within the cytoplasm of HNSCC cells (Fig. 4F). To confirm this interaction, the CoIP assays were performed. The results demonstrated an endogenous interaction between PFKP and ERK2 in LIU-LSC-1 cells (Fig. 4G). We also observed that exogenously overexpressed Flag-tagged PFKP interacted with exogenously overexpressed HA-tagged ERK2 in TU177 cells (Fig. 4H). These data suggest that PFKP may interact with ERK2 during the progression of HNSCC.

Based on the findings of their interaction, we further identified the binding regions between PFKP and ERK2. We generated two fragments of PFKP, consisting of amino acids 1-399 (domain 1) and 412–784 (domain 2) as shown in Fig. 4I. Our IP assay showed that only domain 2 interacted with ERK2 (Fig. 4J). Moreover, the molecular docking model predicted specific residues through which domain 2 of PFKP binds to ERK2. These results revealed that residues K753 and L596 of domain 2 are crucial for binding to ERK2 (Fig. 4K). Accordingly, we constructed two PFKP mutants: K753A and L596A. The CoIP assay showed that mutating K753 to A753 destroyed the interaction between PFKP and ERK2, whereas the L596 mutations in PFKP did not significantly affect the interaction between the two proteins (Fig. 4L). Furthermore, when transfected with plasmids carrying the K753 mutation, no significant increase in p-ERK1/2 was observed in cells transfected with wild-type (WT) PFKP plasmids (Fig. 4M). Taken together, our data show that PFKP may directly interact with ERK2, thereby activating the MAPK/ERK pathway.

### PFKP enhances stability of c-Myc protein through activation of the ERK pathway

**Fig. 5:**
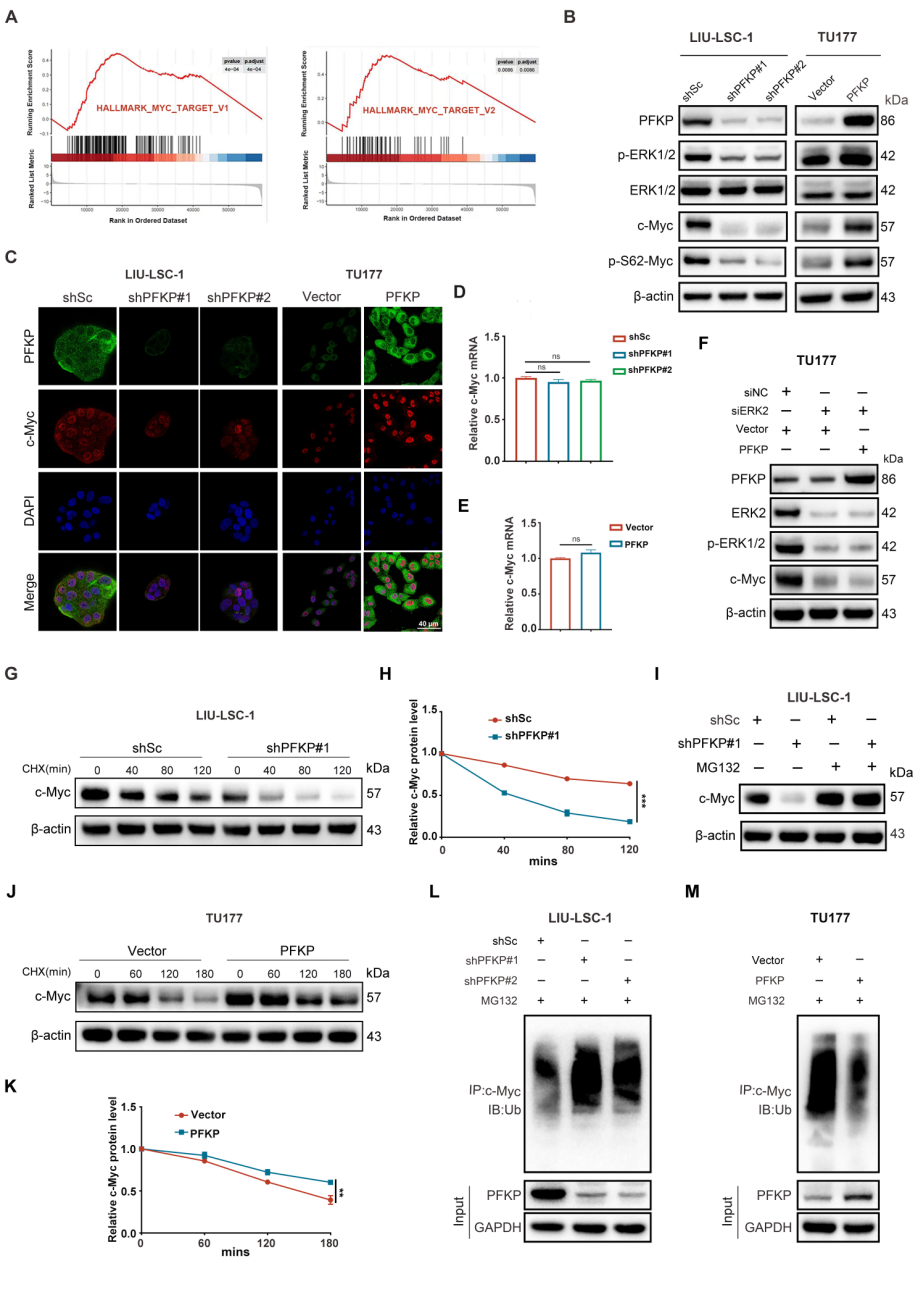
PFKP promotes the ERK-mediated c-Myc stability. (**A**) GSEA was performed in the TCGA-HNSCC cohort to reveal the association between PFKP and the activation of the HALLMARK_MYC_TARGET pathway. (**B**) Protein levels of p-ERK1/2, ERK1/2, c-Myc, and p-S62-Myc were analyzed by the Western blotting in LIU-LSC-1 and TU177 cells. (**C**) Representative immunofluorescence images of LIU-LSC-1 and TU177 cells treated with different treatments as indicated. Scale bars, 40 μm. (**D**-**E**) the qRT-PCR was used to detect the mRNA levels of c-Myc in LIU-LSC-1 cells transfected with PFKP shRNA and TU177 cells transfected with overexpressed PFKP, respectively. (**F**) Protein levels of ERK2, p-ERK1/2, and c-Myc were analyzed by the Western blotting in TU177 cells. (**G** and **H**) Effect of protein synthesis inhibitor CHX (20 µM) on c-Myc stability in PFKP-depleted LIU-LSC-1 cells over time. Protein expression of PFKP and c-Myc stability by Western blotting (left) and semi-quantification (right). (**I**) The Western blotting was preformed to assess the protein levels of c-Myc in knockdown of PFKP with addition of MG132. (**J** and **K**) Effect of CHX on c-Myc stability in PFKP-overexpressing TU177 cells. (L and M) c-Myc polyubiquitination was detected by anti-Ub immunoblotting in PFKP shRNAs LIU-LSC-1 cells (**L**) and LvPFKP TU177 cells (**M**), respectively

The ERK pathway is well-established in regulating the stability of c-Myc by directly phosphorylating c-Myc at S62 [39, 40]. Given the GSEA data from TCGA which showed that enrichment of genes targeted by the Myc signaling in the PFKP-highly expressed groups in HNSCC (Fig. 5A), we hypothesized that PFKP binds to ERK2 and enhances ERK-mediated stability of c-Myc. Consistent with p-ERK1/2 levels, we observed a significant reduction in the levels of c-Myc and p-S62-Myc in the PFKP-knockdown LIU-LSC-1 cells (Fig. 5B, left panel). Conversely, overexpression of WT PFKP led to an upregulation of c-Myc and p-S62-Myc in the TU177 cells (Fig. 5B, right panel). The positive regulation of c-Myc by PFKP was confirmed by the IF assay (Fig. 5C). Additionally, the qRT-PCR analysis further revealed that the regulation of c-Myc by PFKP was not at the mRNA transcription level (Fig. 5D and E). Overexpression of PFKP did not increase c-Myc levels when ERK2 was knocked down in the TU177 cells (Fig. 5F). These findings prompted us to investigate whether PFKP could regulate the stability of c-Myc. Our CHX chase analysis revealed a significant acceleration of c-Myc degradation in PFKP-knockdown LIU-LSC-1 cells compared to that in the control cells (Fig. 5G and H). Furthermore, pretreatment with MG132, a specific 26 S proteasome inhibitor, inhibited the accelerated degradation of c-Myc caused by PFKP depletion (Fig. 5I). Conversely, when PFKP was overexpressed in TU177 cells, it decelerated the degradation of c-Myc protein (Fig. 5J and K). Subsequent ubiquitination assays showed that PFKP depletion promoted the ubiquitination and degradation of c-Myc, while overexpressing PFKP inhibited these processes (Fig. 5L and M). These findings support the conclusion that PFKP may interact with ERK2, leading to the upregulation of c-Myc through suppression of ubiquitin-mediated degradation of c-Myc.

### c-Myc contributes to PFKP-mediated HNSCC progression

**Fig. 6:**
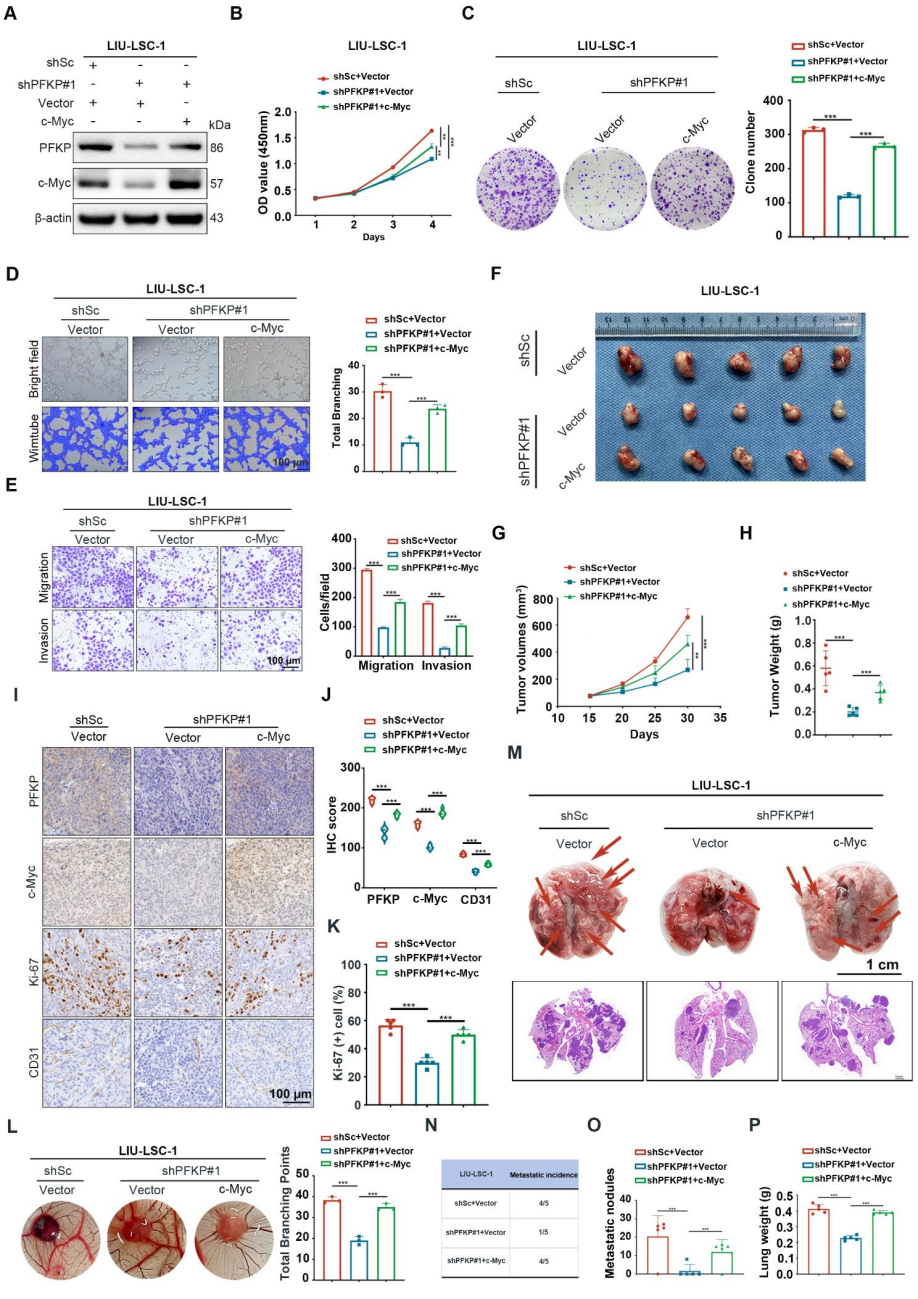
The c-Myc-mediated function of PFKP promotes proliferation, angiogenesis, and metastasis of HNSCC cells. (**A**) shPFKP LIU-LSC-1 cells were infected with lentiviruses harboring a vector encoding human c-Myc or the empty vector. The cell lysates were subjected to immunoblotting. (**B**) and (**C**) Cell proliferation (**B**) and colony formation (**C**) assays were carried out using cells as described in (A). Representative images (left) and corresponding quantification (right) of survival colonies are displayed. (**D**) For tube formation assays, LIU-LSC-1 cells (3.5 × 10^4^) were mixed with conditioned media and incubated for 12 h on Matrigel. Representative micrographs of tube formation assay (left panel). Scale bars, 100 μm. Quantifications of total branching points per microscopic field from three independent experiments were analyzed by WimTube (right panel). (**E**) Cell migration and invasion abilities of the indicated cells were measured by transwell assays (left panel: representative images; right panel: statistical analysis). Scale bars, 100 μm. (**F**-**K**) The indicated cells were inoculated subcutaneously into nude mice, followed by monitoring tumor growth. Tumor images (**F**), tumor volumes at the indicated times (**G**), tumor weight (**H**), representative IHC images of PFKP, c-Myc, CD31, and Ki-67 staining of subcutaneous xenograft tissues (**I**), and the quantitative analysis results of IHC (**J**-**K**). **P < 0.01; ***P < 0.001. Scale bars, 100 μm. (**L**) The indicated cells were subjected to CAM assays. Representative images (left panel) and statistical analysis (right panel) are shown. (**M**-**P**) The indicated LIU-LSC-1 cells were injected into nude mice via the tail vein (*n* = 5 mice/group). (**M**) Representative images of lungs (Scale bar, 1 cm) and H&E-stained lung sections (Scale bar, 1 mm) showing metastatic lesions generated from the indicated cells after tail vein injections. (**N**) The number of metastatic lung nodules. (**O**) Lung metastatic nodules of all animals. (**P**) Lung weight of all animals. Error bars represent the mean ± SD of triplicate technical replicates. ***P < 0.001

To investigate whether c-Myc is involved in PFKP-mediated HNSCC progression, we overexpressed c-Myc in PFKP-knockdown LIU-LSC-1 cells (Fig. 6A). As depicted in Fig. 6B and C, restoring c-Myc reversed the effects of PFKP suppression on LIU-LSC-1 cell proliferation. Furthermore, c-Myc overexpression weakened the inhibitory effect of PFKP knockdown on angiogenesis, migration, and invasion (Fig. 6D and E). In a parallel experiment, we transduced a lentiviral vector expressing shRNA targeting c-Myc into PFKP-overexpressing TU177 cells (Supplementary Fig. 5A). The accelerated cell proliferation, migration, invasion, and angiogenesis driven by PFKP overexpression were inhibited by c-Myc depletion in TU177 cells (Supplementary Fig. 5B-E). Taken together, these data underscore the pivotal role of PFKP-mediated c-Myc expression in the progression of HNSCC.

The role of PFKP-mediated c-Myc expression in HNSCC progression, growth, angiogenesis, and metastasis was further analyzed using a subcutaneous xenograft tumor model in vivo, a CAM assay, and a tail vein lung cancer metastasis mouse model. Consistent with our in vitro results, c-Myc re-expression abolished the suppressive effects of PFKP depletion on xenograft growth (Fig. 6F-K) and tumor angiogenesis (Fig. 6L). The c-Myc overexpression partially rescued the decreased number of pulmonary metastatic nodules in mice injected with PFKP-knockdown LIU-LSC-1 cells. This rescue effect was evident upon macroscopic observation, H&E staining of lung tissues, and measurement of lung weights in tumor-bearing animals (Fig. 6M-P). These results support the notion that PFKP may promote HNSCC progression, growth, and metastasis through enhanced c-Myc expression.

#### c-Myc promotes transcription of PFKP in HNSCC

**Fig. 7:**
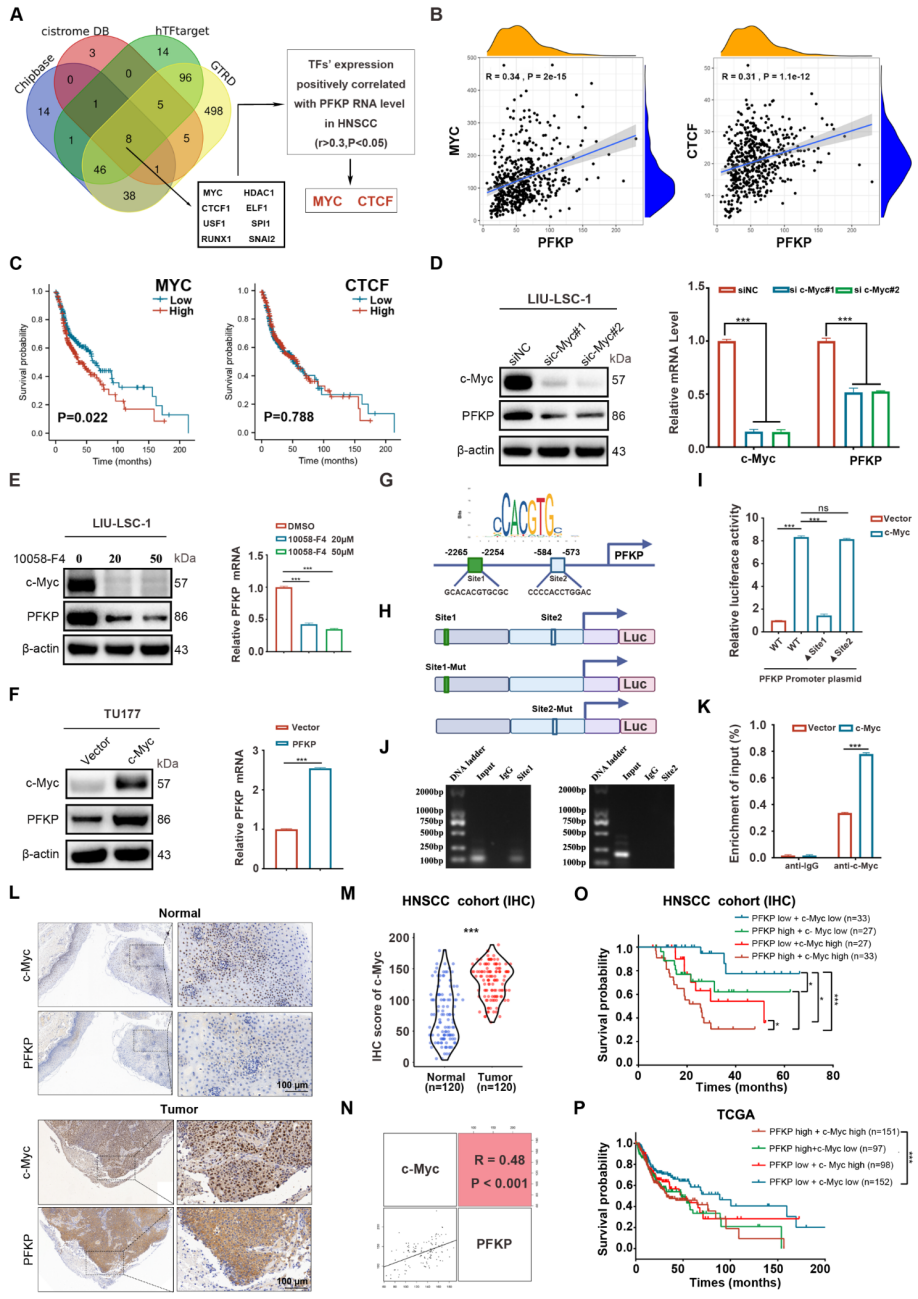
c-Myc stimulates transcription of the *PFKP* gene (**A**) Venn diagram of transcription factors (TFs) positively correlated with the PFKP expression levels in TCGA-HNSCC. (**B**) Pearson’s correlation analysis of PFKP with MYC and CTCF using the TCGA cohort. (**C**) The K-M analysis on the association between MYC or CTCF mRNA levels and OS in TCGA. (D) LIU-LSC-1 cells expressing siRNAs targeting c-Myc or control. Western blotting and qRT-PCR were used to measure protein and mRNA levels. (**E**) LIU-LSC-1 cells were treated with 20 µM or 50 µM 10,058-F4 for 24 h. The samples were subjected to Western blotting and qRT-PCR analyses. (**F**) PFKP expression detected in the cells by Western blotting and qRT-PCR. (**G**) Schematic of the c-myc-binding site on human *PFKP* gene. (**H**) HEK-293T cells co-transfected with the indicated promoter constructs and pGL3-c-Myc or empty pGL3, plus the internal control plasmid pRL-TK. (**I**) Luciferase activity measured 24 h post-transfection. (**J**) HIF-1α antibody-immunoprecipitated DNA from these cells was amplified and quantified by qRT-PCR for NBR and PBR regions. (**K**) The data are plotted as the ratio of immunoprecipitated DNA subtracting nonspecific binding to IgG vs. total input DNA (%). (**L**) Representative IHC images of c-Myc and PFKP staining of the normal and tumor tissues. Scale bar, 100 μm. (**M**) H-score statistical analysis of c-Myc and PFKP IHC staining of normal and tumor tissues. (**N**) Correlation between c-Myc and PFKP expression of tumor tissues. The square in the upper right corner shows the Pearson correlation value between the indicated genes. The scatterplot matrix fitted trend lines for the indicated genes are shown in the square in the lower left corner. (**O**-**P**) K-M curves of OS stratified by PFKP and c-Myc in TCGA-HNSCC (**O**) and our cohort (**P**). Error bars represent the mean ± SD of triplicates. *P < 0.05, **P < 0.01, ***P < 0.001

To investigate the underlying mechanism of elevated PFKP in HNSCC progression, we performed bioinformatics analysis using four public databases. Eight candidate transcription factors (TFs), including c-Myc, CTCF1, ELF1, HDAC1, RUNX1, SNAI2, SPI1, and USF1 were screened (Fig. 7A). To refine the selection of candidate TFs, we analyzed the correlation of mRNA levels of these TFs with PFKP using the TCGA-HNSCC data and found that two TFs (c-Myc and CTCF) exhibited a positive correlation with PFKP (*r* > 0.3, *P* < 0.05; Fig. 7B and Supplementary Fig. 6A). Furthermore, the K-M survival analysis demonstrated that HNSCC patients with high c-Myc mRNA expression had worse OS than those with low expression, while CTCF mRNA expression did not show a significant correlation with the OS of HNSCC (Fig. 7C). Additionally, the ChIP-seq data and RNA-seq data from public databases (GSE138295, GSE126739) revealed that c-Myc has a significant signal in the promoter region of *PFKP* (Supplementary Fig. 6B), and the PFKP mRNA levels were significantly reduced after reduction of c-Myc in lymphoma cells (Supplementary Fig. 6C). Therefore, it is likely that PFKP may be regulated by c-Myc.

As shown in Fig. 7D and E, both genetic and pharmacological inhibition of c-Myc led to a significant reduction in PFKP expression. Conversely, the overexpression of c-Myc led to increased expression of PFKP at both the protein and mRNA levels (Fig. 7F). These findings further substantiate the role of c-Myc in the regulation of PFKP.

To further explore the mechanism underlying the regulation of PFKP by c-Myc, the promoter region (-2500 to + 200) of human *PFKP* was analyzed using JASPAR (https://jaspar.genereg.net), which predicted two possible binding regions (Site 1, -2265 GCACACGTGCGC − 2254; Site 2, -584 CCCCACCTGGAC − 573) of c-Myc (Fig. 7G and H). We then cloned the human *PFKP* gene promoter into the pGL3 luciferase reporter and evaluated the effect of c-Myc on the promoter activity. The promoter activity of the WT *PFKP* promoter construct was enhanced by c-Myc overexpression. Mutation of Site 1 abolished the stimulatory effect of c-Myc, whereas mutation of Site 2 showed only a slight effect on c-Myc-induced *PFKP* promoter activity (Fig. 7I). The ChIP-PCR assay confirmed the enrichment of c-Myc in Site 1, further suggesting that this site may be critical for the transcription of *PFKP* (Fig. 7J). Moreover, the ChIP-qRT-PCR assay demonstrated an increased occupancy of c-Myc on the *PFKP* promoter in the c-Myc-overexpressing cells (Fig. 7K). Based on these results, we conclude that c-Myc may transcriptionally upregulate PFKP through direct binding to its promoter.

We then examined the expression of c-Myc in tumor and adjacent normal tissues of HNSCC patients by IHC staining. Consistent with PFKP, the expression of c-Myc was higher in the cancer tissues than in their paired normal tissues (Fig. 7L and M). Pearson correlation analyses further revealed a positive correlation between PFKP and c-Myc (*r* = 0.48, *P* < 0.001; Fig. 7N). The K-M survival analysis on our cohort demonstrated that HNSCC patients with high expression of both PFKP and c-Myc had a significantly worse OS compared to those with low expression of PFKP and c-Myc (Fig. 7O). Furthermore, similar results were observed in HNSCC patients in the TCGA dataset (Fig. 7P). Thus, we demonstrate that c-Myc may upregulate the transcription of PFKP by binding to the promoter of the *PFKP* gene, and both PFKP and c-Myc may contribute to the prognosis of HNSCC.

### Targeting PFKP and c-Myc inhibits HNSCC tumor progression

**Fig. 8:**
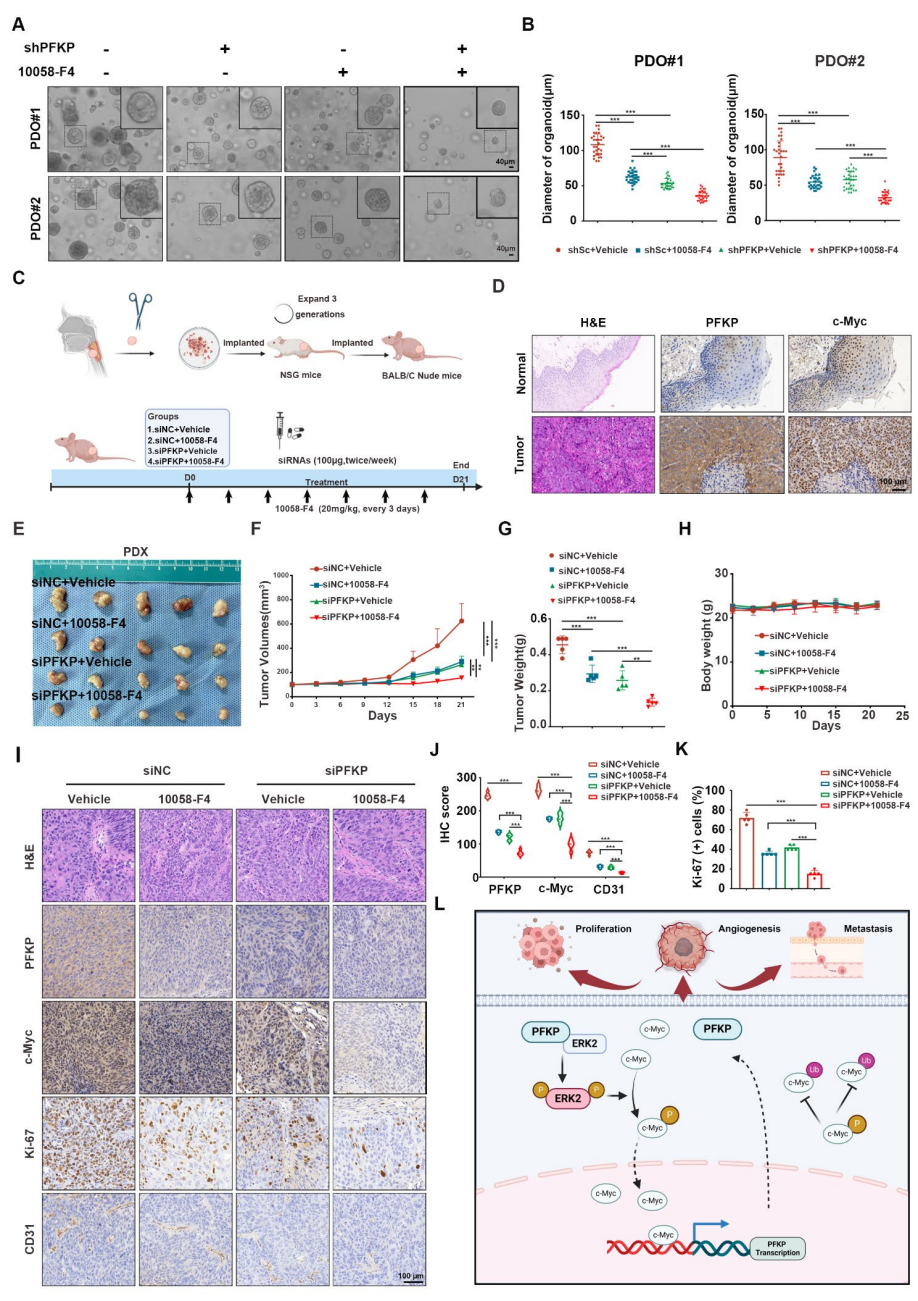
The co-treatment of both PFKP inhibition and 10,058-F4 has inhibitory effect on growth in PDO and PDX models. (**A**) Effects of sh-PFKP and 10,058-F4 on the viability of PDO cells (scale bar, 40 μm). (**B**) Measurement of the growth of PDO in response to knocking down PFKP and 10,058-F4. ***P < 0.001. (**C**-**I**) Effects of 10,058-F4 combined with PFKP siRNAs on LIU-LSC-1 xenograft tumor growth. (C) Schematic illustration of experimental design and timeline. (**D**) IHC staining of PFKP and c-Myc in paracancerous and HNSCC tissues (scale bar, 100 μm). Tumor images (**E**), tumor volume (**F**), tumor weight (**G**), and body weight (**H**) are shown. **P < 0.01, ***P < 0.001. (**I**) Representative IHC images of PFKP, c-Myc, Ki-67, and CD31 staining for indication of the PDX tumors (scale bar, 100 μm). (**J**-**K**) the quantitative analysis results for PFKP, c-Myc, CD31 and Ki-67. Error bars indicate mean ± SD. ***P < 0.001. (L) Schematic diagram illustrating the feedback loop formed by PFKP and c-Myc that promotes HNSCC proliferation, angiogenesis, and metastasis. Schematic was developed with BioRender (www.biorender.com)

The observation that a feedback loop between PFKP and c-Myc contributes to HNSCC tumor progression, coupled with the finding that patients with high expression of both PFKP and c-Myc have worse survival outcomes, prompted us to investigate whether co-inhibition of PFKP and c-Myc using the inhibitor 10,058-F4 could synergistically impede the progression of HNSCC. We first assessed the therapeutic effects of the combination of PFKP with c-Myc inhibition using HNSCC PDO models. As depicted in Fig. 8A and B, the co-inhibition of PFKP and c-Myc more effectively suppressed PDO growth than either inhibition treatment alone. Furthermore, we injected LIU-LSC-1 cells into nude mice to establish a subcutaneous tumor model (Supplementary Fig. 7A). The mice were treated with PFKP siRNA or siNC along with intraperitoneal injection of 10,058-F4 or a vehicle control. The combination of PFKP siRNA and 10,058-F4 led to a stronger inhibitory effect on LIU-LSC-1 xenografts than either single agent alone, as shown by tumor volume and weight (Supplementary Fig. 7B-D). The loss of body weight in the treated mice was not observed (Supplementary Fig. 7E). The IHC staining and scoring revealed that the expressions of Ki-67 and CD31 were decreased in the treatment group compared with those in the control group, which was consistent with the tumor burdens in the different groups (Supplementary Fig. 7F-H).

The fresh HNSCC tumors (HPV negative) with high expression of PFKP and c-Myc were chosen to establish the PDX models (Fig. 8C and D, and Supplementary Fig. 8A). Consistent with our CDX findings, co-treatment with PFKP siRNAs and 10,058-F4 more effectively suppressed PDX tumor growth compared to either treatment alone (Fig. 8E-G). Crucially, no weight loss was observed in the treated mice (Fig. 8H). The successful reduction of PFKP in tumors by siRNAs was verified using the IHC and Western blotting analyses (Fig. 8I-K and Supplementary Fig. 8B). The IHC staining of Ki-67 and CD31 in the tumors of the PDX models showed a similar pattern to that of the CDX models. Taken together, our results show that the combination of PFKP with c-Myc inhibition may be a promising strategy for a more effective treatment of HNSCC.

## Discussion

In the current study, we demonstrate that PFKP levels are elevated in HNSCC and are associated with worse OS in HNSCC patients. PFKP may contribute to HNSCC progression by enhancing ERK-mediated c-Myc stability. Furthermore, the accumulation of c-Myc promotes the transcription of PFKP, forming a positive feedback loop that intensifies HNSCC proliferation, angiogenesis, and metastasis. Finally, the combined inhibition of PFKP and c-Myc may yield synergistically anti-tumor effects on HNSCC progression.

PFKP, a glycolytic rate-limiting enzyme, exhibits abnormal overexpression in multiple cancers and plays a critical role in tumorigenesis. For instance, studies have shown that PFKP is highly expressed in lung cancer tissues, and its silencing resulted in a significant reduction in cell growth, and impaired colony-forming ability [12]. Sha et al. found that PFKP levels are higher in hepatocellular carcinoma (HCC) tissues than in normal hepatic tissues, and silencing PFKP decreases HCC cell proliferation [41]. In the present study, we propose that PFKP plays a crucial role in HNSCC progression based on several pieces of evidence. Firstly, we observed an upregulation of PFKP expression in HNSCC tissues and cell lines, with elevated PFKP levels associated with poorer survival in HNSCC patients. Secondly, through in vitro experiments, we revealed that PFKP promotes the proliferation, angiogenesis, migration, and invasion of HNSCC cells. Thirdly, the oncogenic role of PFKP was confirmed by organoids and PDX models in HNSCC. Our findings, along with others [11, 42], indicate that PFKP may be a pivotal factor in HNSCC tumorigenesis and progression, making it a potential therapeutic target for HNSCC.

PFKP promotes the malignant progression of various tumors through the regulation of aerobic glycolysis [10, 43, 44]. Emerging evidence underscores its involvement in promoting cell survival and cancer cell proliferation through mechanisms independent of glycolysis. For example, Chen et al. revealed a critical non-glycolysis-related function of PFKP, i.e., regulating long-chain fatty acid oxidation via AMP-activated protein kinase, which promotes lung cancer cell survival under glucose starvation [45]. Wang et al. provided a new mechanistic insight into the regulation of PD-L1 expression by a non-metabolic function of PFKP in tumor cells [46]. In this study, through integrated analysis of RNA-seq data from PFKP-knockdown cells, mass spectrometry data from PFKP-immunoprecipitated proteins, and subsequent functional validation experiments, we demonstrate that PFKP may directly bind to ERK2, thus activating the MAPK/ERK pathway in HNSCC cells. This finding not only broadens our understanding of the non-glycolytic mechanisms of PFKP in tumorigenesis but also explains why the MAPK/ERK signaling pathway is highly activated in HNSCC. Indeed, the connection between PFKP and the MAPK pathway has been implicated in previous studies. For instance, two independent studies have shown that reduced PFKP expression can attenuate p-ERK1/2 levels in triple-negative breast cancer cell lines and ovarian cancer cell lines [47, 48]. Therefore, it is likely that depletion of PFKP disrupts the interaction between PFKP and ERK2, thereby inhibiting the activation of the MAPK/ERK pathway in these cells. However, the specific mechanism by which PFKP enhances ERK1/2 activity remains unclear. It is well established that EGFR is a critical upstream regulator of ERK1/2 [49]. We discovered that PFKP interacts with EGFR, activating ERK1/2 in an EGFR-dependent manner in HNSCC cells, as shown in Supplementary Fig. S9A-D. Thus, it is likely that the interaction between PFKP, EGFR, and ERK2 may contribute to the activation of ERK1/2 by EGFR. We aim to further explore this hypothesis in our future studies.

c-Myc functions as a master regulator of multiple biological processes, primarily acting as a transcription factor that may directly or indirectly govern the expression of many genes [50]. The activation of c-Myc in cancers can occur through multiple signaling pathways. For example, Wnt binding stabilizes the transcription factor β-catenin, facilitating its nuclear entry to regulate Wnt pathway target genes, such as c-Myc [51]. Inhibitors of the PI3K/AKT/mTOR signaling pathway suppress c-Myc translation, thereby reducing tumor levels of c-Myc in a mouse tumor model [52]. Mitogenic pathways, such as RAS-MEK-ERK signaling, can elevate P-S62 c-Myc levels, consequently increasing c-Myc stability [53, 54]. In our current study, we found that PFKP enhances the stability of c-Myc via ERK. Furthermore, analysis of our HNSCC tumor samples confirmed a positive correlation between PFKP and c-Myc expression. Moreover, GSEA enrichment analysis of RNA-sequencing from data TCGA revealed that Myc signaling pathway was enriched in the PFKP-highly expressed group in HNSCC and various other cancer types (Fig. 5A and Supplementary Fig. 10). Thus, we conclude that c-Myc could partially mediate the function of PFKP in PFKP-associated HNSCC. Additionally, the link between PFKP and c-Myc has also been reported in other cancer types. For example, previous report indicated that PFKP serves as a nucleocytoplasmic shuttling protein, with nuclear PFKP capable of interacting with c-Myc, thereby stabilizing it and enhancing its activity in T-cell acute lymphoblastic leukemia [55]. Our findings further validate that PFKP can enhance the stability of the c-Myc protein. However, the results from IF analysis, IHC analyses, and nuclear-cytoplasmic fractionation (Supplementary Fig. 11) indicate that PFKP is predominantly located in the cytoplasm within HNSCC cells and tissues. Therefore, the involvement of PFKP in nuclear functions is likely to be ruled out in HNSCC. Furthermore, PFKP has been reported to enhance the nuclear translocation and activation of β-catenin, further augmenting the expression of its downstream gene, c-Myc, in human glioblastoma cells [56]. In contrast, our current study demonstrates that PFKP may positively regulate c-Myc expression but do not affect its transcriptional levels in HNSCC cells. It is more likely that other regulatory mechanisms are involved in the regulation between PFKP and c-Myc across different tumor types. Moreover, several studies have demonstrated that PFKP may promote angiogenesis in various tumors by upregulating the expression of VEGF [33, 34, 56], with c-Myc serving as a regulator of VEGF [57, 58]. Indeed, overexpression of c-Myc may counterbalance the decrease in VEGFA after PFKP knockdown, as shown in Supplementary Fig. 12. Therefore, we concluded that PFKP might likely enhance VEGF expression through c-Myc, thereby promoting angiogenesis in HNSCC.

Given the role of PFKP overexpression in human malignancies, it is necessary to explore the mechanisms involved in controlling PFKP regulation. Recent research has identified several transcription factors that regulate the transcription of the *PFKP* gene. For example, KLF4 activates the transcription of the *PFKP* gene by directly binding to the *PFKP* promoter, playing a critical role in cell proliferation in breast cancer cells [8]. PFKP is also subject to regulation by HIF-1α at the translational level, promoting cancer cell invasion [59]. Moreover, other transcription factors, such as Snail and STAT3, have been reported to participate in the transcription regulation of PFKP in various types of cells [60, 61]. PFKP may also undergo post-transcriptional regulation by the RNA-binding protein fat mass and obesity-associated protein (FTO) [44]. In our present study, bioinformatics analysis and validation experiments found that c-Myc may directly bind to the promoter region of *PFKP*, thereby enhancing its transcription levels. These results indicate that PFKP could promote the expression levels of c-Myc by activating the MAPK/ERK pathway. The accumulation of c-Myc, in turn, may promote the expression levels of PFKP, forming a positive feedback loop that may drive tumorigenesis in HNSCC. Finally, HNSCC patients with the high expression of both PFKP and c-Myc typically had poorer survival than those with low expression levels of PFKP and c-Myc. Therefore, we speculate that simultaneous inhibition of PFKP and c-Myc may have a highly therapeutic potential in HNSCC patients. Indeed, we found that targeting PFKP with siRNA or shRNA in combination with a c-Myc inhibitor had a potent anti-tumor effect in three preclinical tumor models, including the LIU-LSC-1 CDX, HNSCC PDO, and HNSCC PDX models, respectively.

HPV infection is considered a key risk factor for HNSCC and is also associated with patients’ prognosis [4]. To further explore the role of PFKP in HNSCC survival outcome in relation to HPV status, we used the TCGA dataset for such analysis. Our results indicated no difference in PFKP expression based on tumor location (Supplementary Fig. 13A) or HPV status (Supplementary Fig. 13B). Furthermore, PFKP expression were not significantly associated with OS, Disease- Specific Survival (DSS), and Progression-Free interval (PFI), respectively, in either HPV- or HPV + group) (Supplementary Fig. 13C). The lack of significance could be partially due to small number of cases in each subgroup in the TCGA dataset. A large study is warranted for our further research.

In conclusion, this study demonstrates a novel mechanism in which PFKP and c-Myc form a positive feedback loop to promote the malignant progression of HNSCC (Fig. 8L). The simultaneous inhibition of PFKP and c-Myc may be a novel therapeutic strategy for future treatment of HNSCC.

### Electronic supplementary material

Below is the link to the electronic supplementary material.

Supplementary Material 1. IHC staining of PFKP in various tissue samples, with intensity scores ranging from 0 to 3. Scale bars: 100 µm.

Supplementary Material 2. Assessment of HPV status and PFKP levels in PDO samples and donor tumor tissues. (A) Gel electrophoresis of PCR amplification products with primer pairs MY09/MY11 and GP5+/GP6+ for detecting HPV DNA in Hela cells (positive control), tumor samples, and ddH2O (negative control). (B) IHC staining showing PFKP expression in donor tumor tissue samples. Scale bars: 50 µm. (C) Western blotting analysis for PFKP levels in tumor tissues and PDOs. (D) Manipulation of PFKP expression through knockdown and overexpression in two individual organoids, with subsequent detection of PFKP by Western blotting.

Supplementary Material 3. Series of experiments investigating the roles of PFKP in various cellular processes in HNSCC cells. (A) Relative PFK-1 activity. (B) Relative glucose uptake. (C) lactate production after silencing PFKP. (D) Flow cytometry analysis of cell cycle distribution in LIU-LSC-1 cells after PFKP knockdown, showing changes in the percentage of cells in G1, S, and G2 phases compared to control. (E) Results of apoptosis analysis. (F) Detection of VEGFA expression by Western blotting. (H) Analysis of EMT markers levels by Western blotting. Error bars indicate mean ± SD of triplicate samples. **P<0.01; ***P<0.001.

Supplementary Material 4. Secondary mass spectra image of the unique petides of ERK2, from co-immunoprecipitation (coIP) experiments.

Supplementary Material 5. Role of c-Myc in PFKP-induced proliferation, angiogenesis, migration and invasion in TU177 cells. (A) c-Myc shRNA- or control shRNA (shSc)-expressing lentiviruses were transduced into TU177 cells overexpressing PFKP and control cells. The protein expression of PFKP and c-Myc was detected by Western blotting. The cells were subjected to CCK-8 (B), colony formation (C), tube formation (D), and transwell (E) assays. Error bars indicate mean ± SD of triplicate samples. ***P<0.001.

Supplementary Material 6. c-Myc directly transactivates PFKP expression. (A) Scattergram showing the mRNA expression correlations of PFKP, ELF1, HDAC1, RUNX1, SNAI2, SPI1, and USF1 from TCGA database. (B) Genome browser tracks of c-Myc occupancy in the PFKP loci in SKNSH, SKNAS, or NB69 cells (public dataset: GSE138295). The genome browser map is displayed by IGV software. The brown region in the PFKP promoter is where c-Myc is significantly enriched relative to input. (C) Heatmap showing the effect of c-Myc knockdown on the expression of PFKP compared to that in control scrambled shRNA cells.

Supplementary Material 7. Depletion of PFKP increases sensitivity to 10058-F4 in HNSCC cells. (A) Schematic representation of the treatment regimen for the CDX model, indicating the timeline for siRNA treatments and 10058-F4 administration. (B) Tumor images. (C) Tumor growth curve. (D) Tumor weight was measured after tumor excision. (E) Body weight of mice. (F) Representative IHC staining of PFKP, c-Myc, Ki-67, and CD31 in xenograft tumors. Scale bars: 100 ?m. (G-H) the quantitative analysis results for PFKP, c-Myc, CD31 and Ki-67. Error bars indicate mean ± SD of quintuplicate sample. **P<0.01; ***P<0.001.

Supplementary Material 8. The co-treatment of both PFKP inhibition and 10058-F4 has inhibitory effect on growth in PDX models. (A) Gel electrophoresis image showing PCR amplification products using primer pairs MY09/MY11 and GP5+/GP6+, testing for HPV DNA in Hela cells (positive control), PDX tumor samples, and ddH_2_O (negative control). (B) Tumor tissues derived from each group were subjected to immunoblotting.

Supplementary Material 9. PFKP is involved in EGFR-mediated activation of ERK1/2. (A) immunoprecipitation (IP) assay followed by Western blotting analysis showing the interaction between PFKP and components of the EGFR-ERK1/2 signaling pathway, including EGFR, ERK2, MEK1/2, and RAS in LIU-LSC-1 cells. (B) Western blotting was used to detect the expression of the indicated proteins after knocking down PFKP in LIU-LSC-1 cells or overexpressing PFKP in TU177 cells. (C) LIU-LSC-1 cells transfected with PFKP shRNA were stimulated with or without EGF (50 ng/mL) for 15 min. (D) Western blotting analysis assesses the effects of PFKP overexpression and the application of the EGFR inhibitor Gefitinib (10 ?M for 48 hours) on EGFR and ERK1/2 activation in TU177 cells, and EGF treatment (50 ng/mL for 1 hour).

Supplementary Material 10. PFKP expression positively correlates with the activation of the HALLMARK_MYC_TARGET pathway in multiple human cancer tissues. Gene set enrichment analysis (GSEA) was performed to reveal the association between PFKP and the activation of the HALLMARK_MYC_TARGET pathway. (A) Adrenocortical Carcinoma (B) Breast invasive carcinoma (C) Cholangiocarcinoma (D) Kidney Chromophobe (E) Kidney renal papillary cell carcinoma (F) Pancreatic adenocarcinoma (G) Testicular Germ Cell Tumors (H) Esophageal carcinoma (I) Stomach adenocarcinoma.

Supplementary Material 11. Western blotting analysis detects the subcellular localization of PFKP, phosphorylated ERK1/2 (p-ERK1/2), ERK2, and c-Myc in the cytoplasmic and nuclear fractions of LIU-LSC-1 and TU177 cells. Lamin b1 and GAPDH are included as nuclear and cytoplasmic markers, respectively, to confirm the purity of the fractions.

Supplementary Material 12. Cells were transfected with shRNA specific for PFKP (shPFKP#1) or a scramble control (shSc), and with or without a c-Myc expression vector. The presence of c-Myc, PFKP, and VEGFA proteins was detected using Western blotting.

Supplementary Material 13. Analysis of PFKP expression in HNSCC across various anatomical locations and HPV status, and its association with patient survival outcomes in TCGA dataset. (A). Expression levels of the PFKP gene in three different anatomical locations of HNSCC (larynx, oral cavity, and hypopharynx cancers). (B). Expression of PFKP in HNSCC based on HPV status. Data source: UALCAN website (https://ualcan.path.uab.edu/). (C). Kaplan-Meier survival curves for HNSCC patients based on HPV status and PFKP expression levels. The top row displays OS, DSS, and PFI, respectively, for HPV- patients (80 cases), and the bottom row shows the same metrics for HPV+ patients (33 cases).

Supplementary Material 14: : Supplementary Table 1. Clinical features of 120 HNSCC patients.

Supplementary Material 15: Supplementary Table 5. Differentially expressed genes of the RNA-seq (shPFKP LIU-LSC-1 cells vs. shSc LIU-LSC-1 cells).

Supplementary Material 16: Supplementary Table 6. Differentially expressed genes enriched in KEGG pathway (shPFKP LIU-LSC-1 cells vs. shSc LIU-LSC-1).

Supplementary Material 17: Supplementary Table 7. Enciched GO Pathway of differentially expressed genes (shPFKP LIU-LSC-1 cells vs. shSc LIU-LSC-1).

## Data Availability

No datasets were generated or analysed during the current study.

## Abbreviations

PFKP: Phosphofructokinase-platelet
HNSCC: Head and neck squamous cell carcinoma
CDX: Cell-derived xenograft
PDX: Patient-derived xenograft
PDO: Patient Derived Organoid
PFK-1: Phosphofructokinase 1
PFKL: Phosphofructokinase-liver
PFKM: Phosphofructokinase-muscle
CHX: Cycloheximide
H&E: Hematoxylin and eosin
IHC: Immunohistochemical staining
IF: Immunofluorescence
NOK: Normal oral epithelial cell line
HUVECs: Human umbilical vein endothelial cells
HEK-293T: Human embryonic kidney 293T
qRT-PCR: Quantitative real-time PCR
RNA-seq: RNA sequencing
CCK-8: Cell counting kit-8
CM: Conditioned medium
CAM: Chicken chorioallantoic membrane
CoIP: Co-immunoprecipitation
ChIP: Chromatin Immunoprecipitation
OS: Overall survival
KEGG: Kyoto Encyclopedia of Genes and Genomes
GSEA: Gene set enrichment analysis
DEGs: Differentially expressed genes
ERK: Extracellular regulated protein kinases
TFs: Transcription factors
siRNAs: Small interfering RNAs
MAPK: Mitogen-activated protein kinase
HPV: Human Papillomavirus
