# Supplementary figures and images for: A positive feedback loop between PFKP and c-Myc drives head and neck squamous cell carcinoma progression
Source: Mol Cancer. 2024 Jul 9;23:141. doi: 10.1186/s12943-024-02051-6 (PMC11232239; doi:10.1186/s12943-024-02051-6)

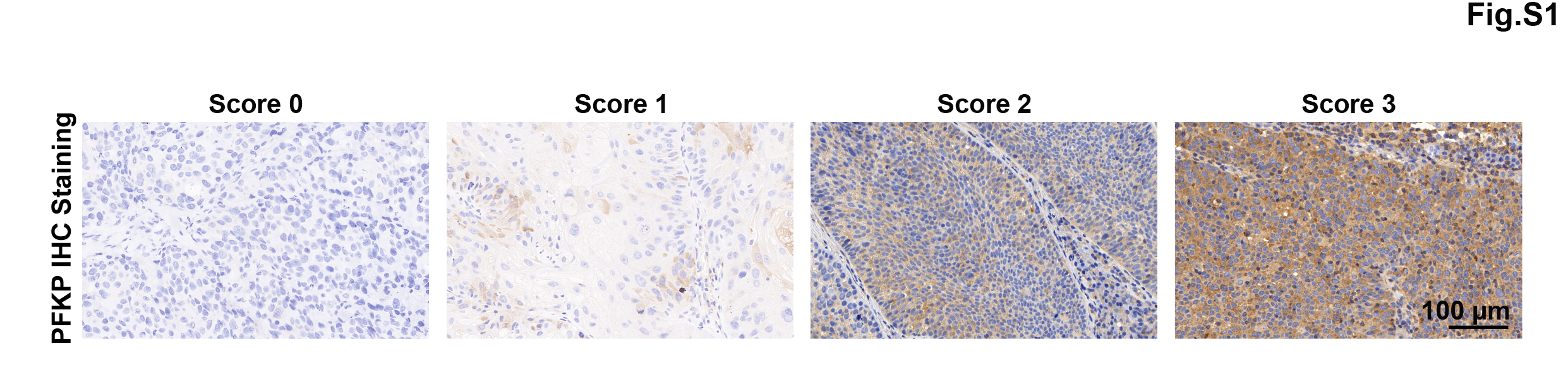

Supplement: Supplementary file 1 — Supplementary Material 1. IHC staining of PFKP in various tissue samples, with intensity scores ranging from 0 to 3. Scale bars: 100 µm. [file 12943_2024_2051_MOESM1_ESM.png]

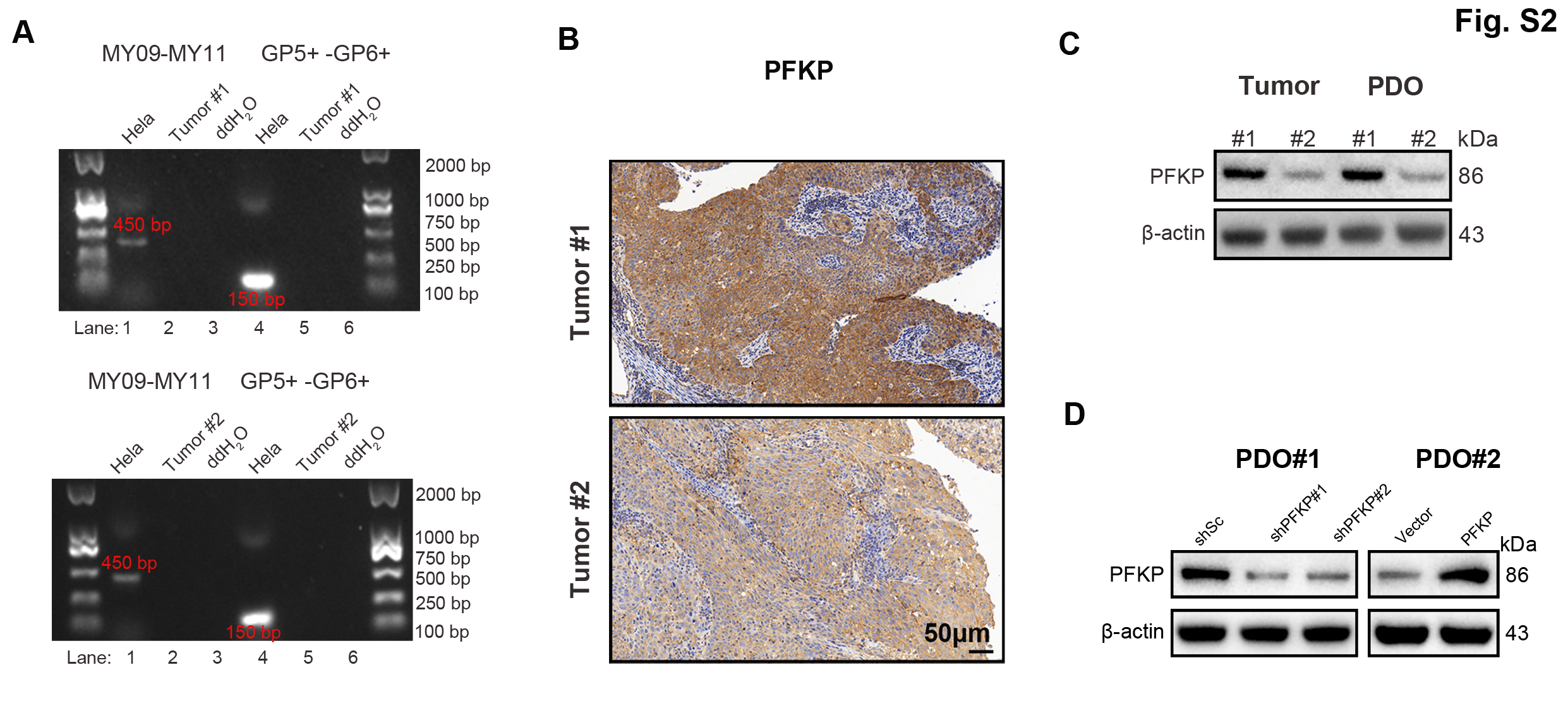

Supplement: Supplementary file 2 — Supplementary Material 2. Assessment of HPV status and PFKP levels in PDO samples and donor tumor tissues. (A) Gel electrophoresis of PCR amplification products with primer pairs MY09/MY11 and GP5+/GP6+ for detecting HPV DNA in Hela cells (positive control), tumor samples, and ddH2O (negative control). (B) IHC staining showing PFKP expression in donor tumor tissue samples. Scale bars: 50 µm. (C) Western blotting analysis for PFKP levels in tumor tissues and PDOs. (D) Manipulation of PFKP expression through knockdown and overexpression in two individual organoids, with subsequent detection of PFKP by Western blotting. [file 12943_2024_2051_MOESM2_ESM.png]

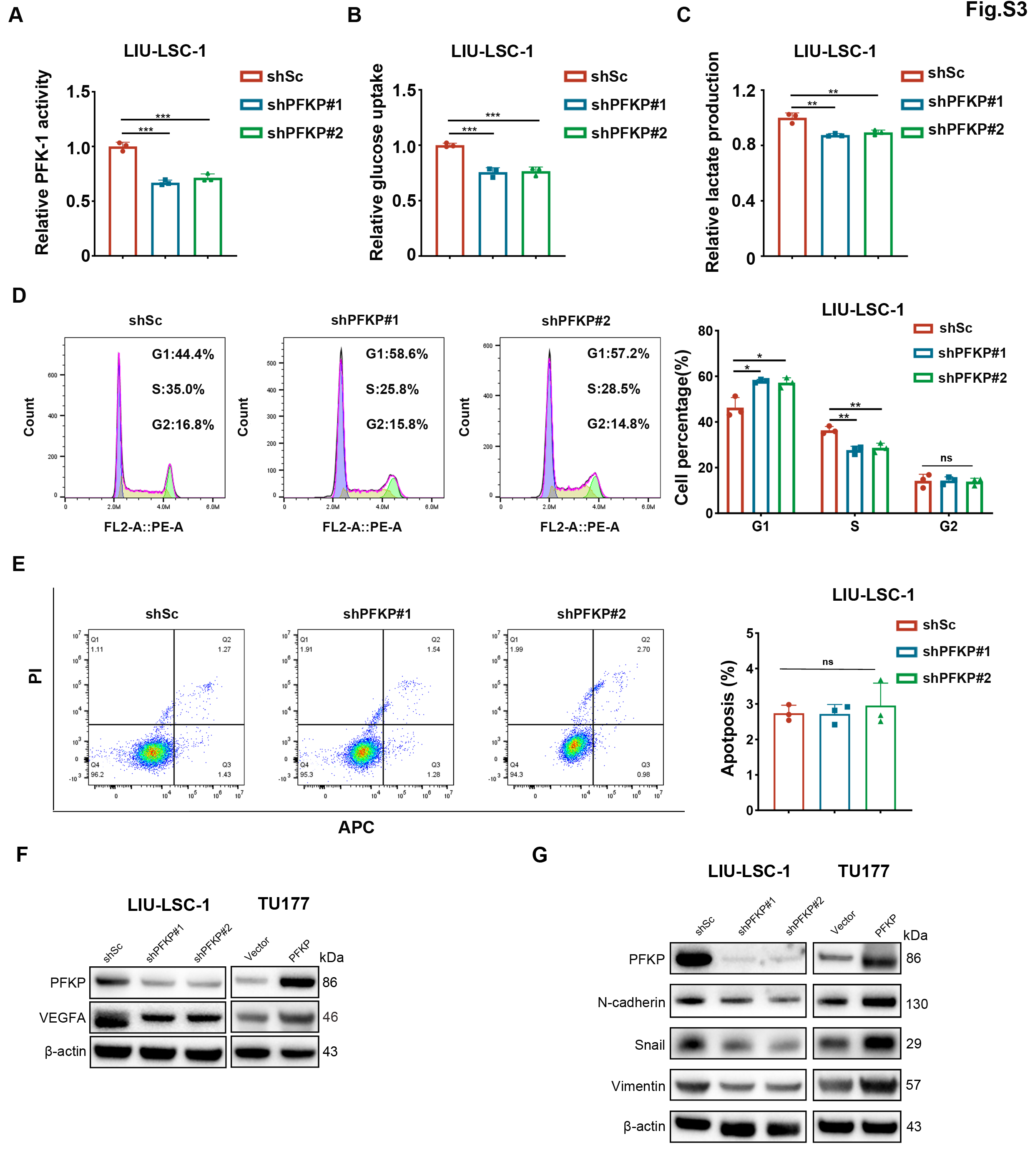

Supplement: Supplementary file 3 — Supplementary Material 3. Series of experiments investigating the roles of PFKP in various cellular processes in HNSCC cells. (A) Relative PFK-1 activity. (B) Relative glucose uptake. (C) lactate production after silencing PFKP. (D) Flow cytometry analysis of cell cycle distribution in LIU-LSC-1 cells after PFKP knockdown, showing changes in the percentage of cells in G1, S, and G2 phases compared to control. (E) Results of apoptosis analysis. (F) Detection of VEGFA expression by Western blotting. (H) Analysis of EMT markers levels by Western blotting. Error bars indicate mean ± SD of triplicate samples. **P<0.01; ***P<0.001. [file 12943_2024_2051_MOESM3_ESM.png]

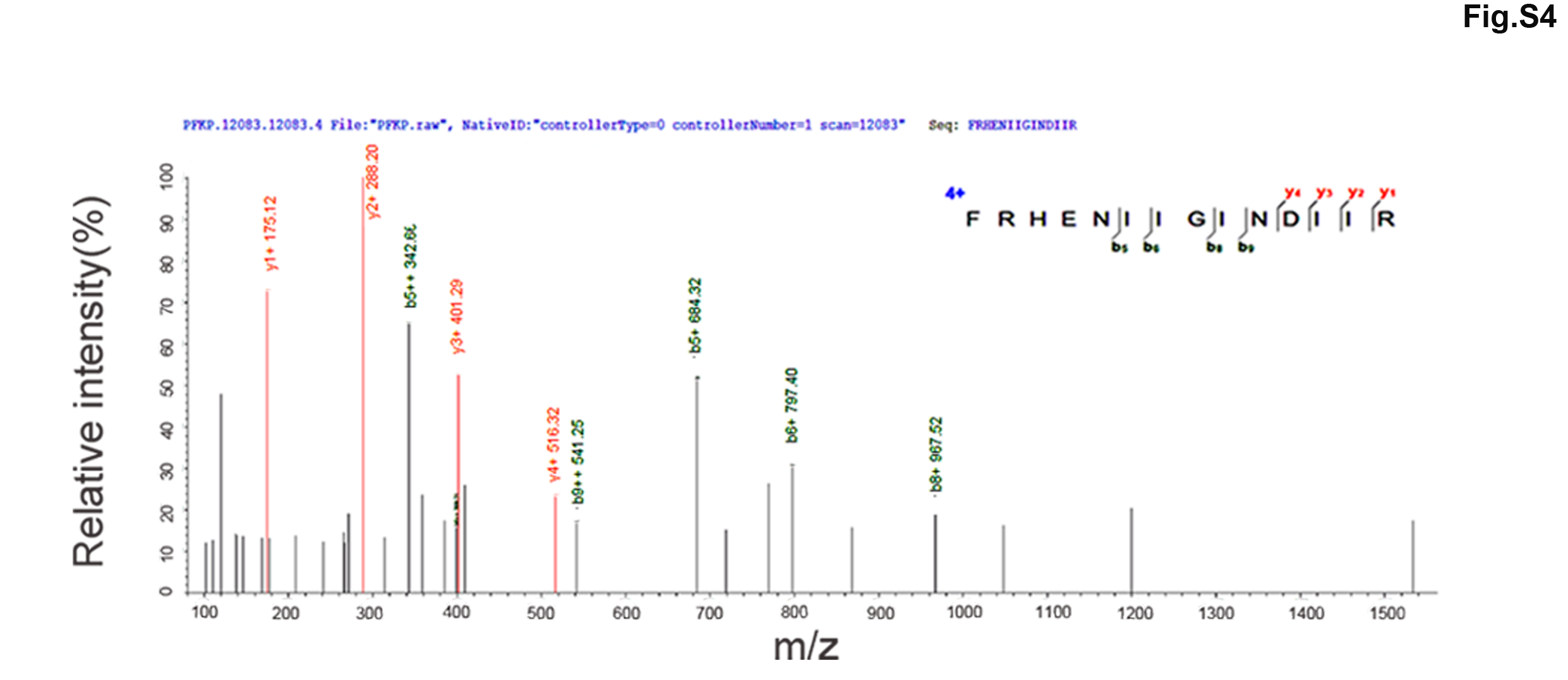

Supplement: Supplementary file 4 — Supplementary Material 4. Secondary mass spectra image of the unique petides of ERK2, from co-immunoprecipitation (coIP) experiments. [file 12943_2024_2051_MOESM4_ESM.png]

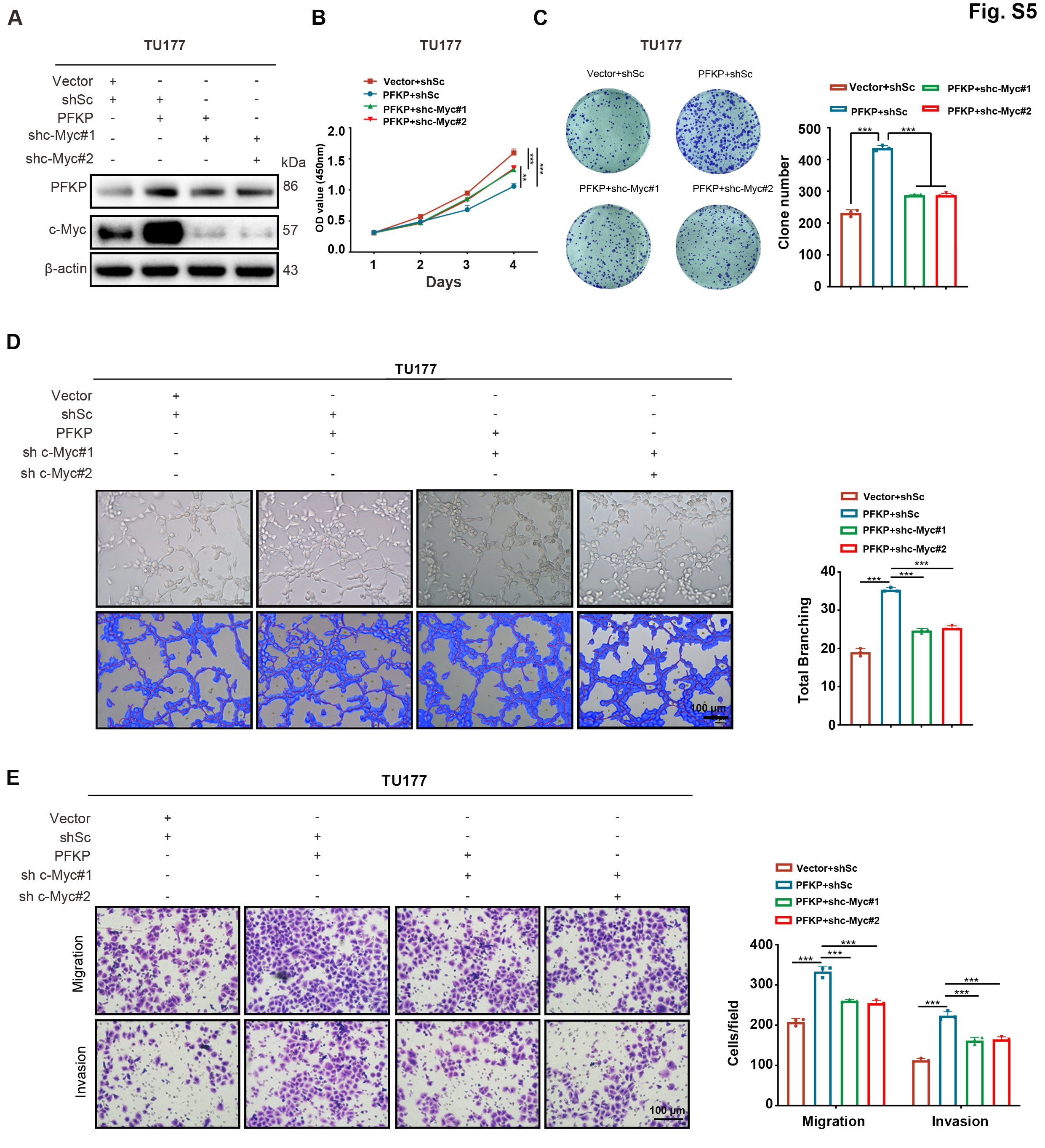

Supplement: Supplementary file 5 — Supplementary Material 5. Role of c-Myc in PFKP-induced proliferation, angiogenesis, migration and invasion in TU177 cells. (A) c-Myc shRNA- or control shRNA (shSc)-expressing lentiviruses were transduced into TU177 cells overexpressing PFKP and control cells. The protein expression of PFKP and c-Myc was detected by Western blotting. The cells were subjected to CCK-8 (B), colony formation (C), tube formation (D), and transwell (E) assays. Error bars indicate mean ± SD of triplicate samples. ***P<0.001. [file 12943_2024_2051_MOESM5_ESM.png]

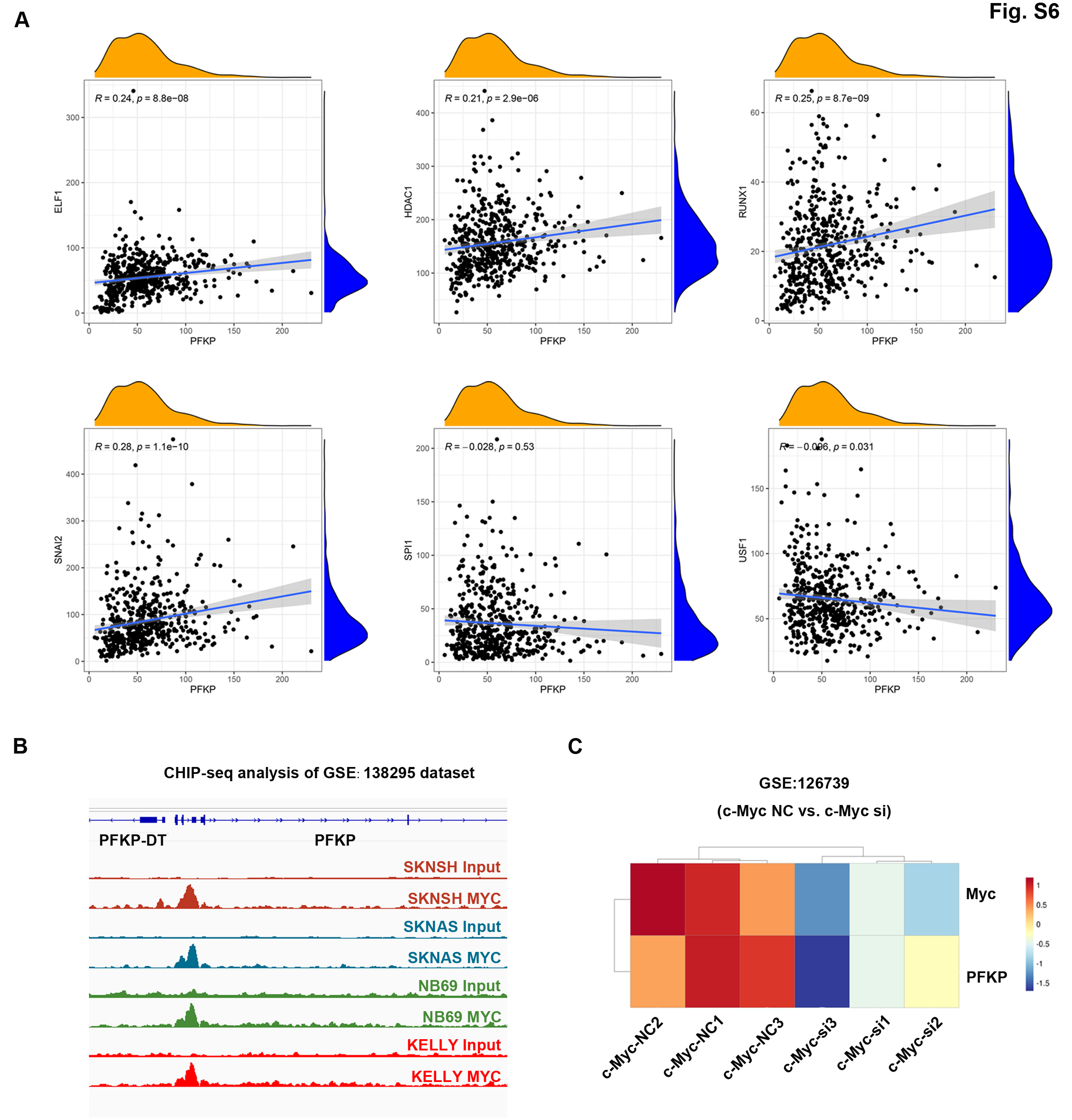

Supplement: Supplementary file 6 — Supplementary Material 6. c-Myc directly transactivates PFKP expression. (A) Scattergram showing the mRNA expression correlations of PFKP, ELF1, HDAC1, RUNX1, SNAI2, SPI1, and USF1 from TCGA database. (B) Genome browser tracks of c-Myc occupancy in the PFKP loci in SKNSH, SKNAS, or NB69 cells (public dataset: GSE138295). The genome browser map is displayed by IGV software. The brown region in the PFKP promoter is where c-Myc is significantly enriched relative to input. (C) Heatmap showing the effect of c-Myc knockdown on the expression of PFKP compared to that in control scrambled shRNA cells. [file 12943_2024_2051_MOESM6_ESM.png]

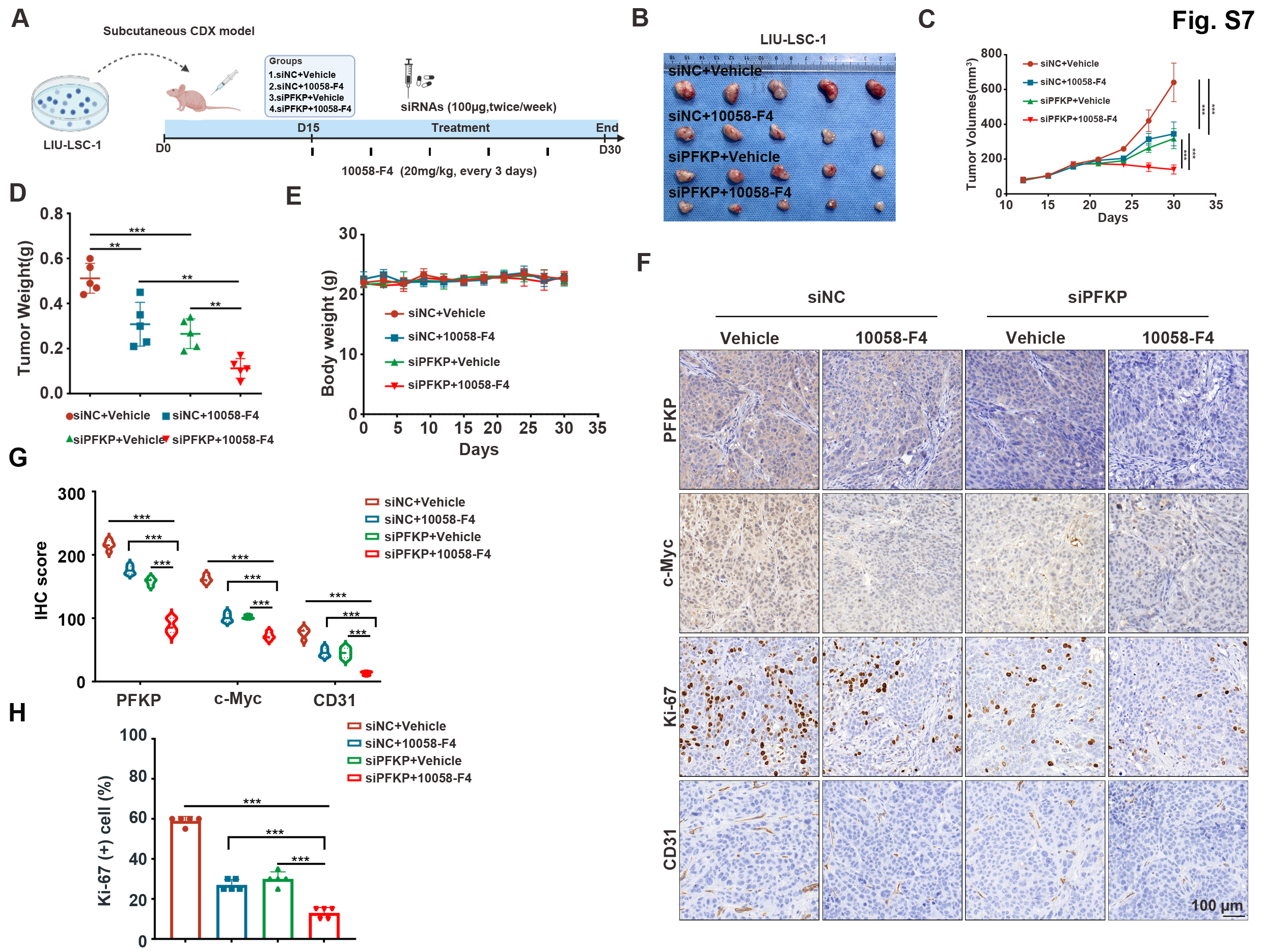

Supplement: Supplementary file 7 — Supplementary Material 7. Depletion of PFKP increases sensitivity to 10058-F4 in HNSCC cells. (A) Schematic representation of the treatment regimen for the CDX model, indicating the timeline for siRNA treatments and 10058-F4 administration. (B) Tumor images. (C) Tumor growth curve. (D) Tumor weight was measured after tumor excision. (E) Body weight of mice. (F) Representative IHC staining of PFKP, c-Myc, Ki-67, and CD31 in xenograft tumors. Scale bars: 100 ?m. (G-H) the quantitative analysis results for PFKP, c-Myc, CD31 and Ki-67. Error bars indicate mean ± SD of quintuplicate sample. **P<0.01; ***P<0.001. [file 12943_2024_2051_MOESM7_ESM.png]

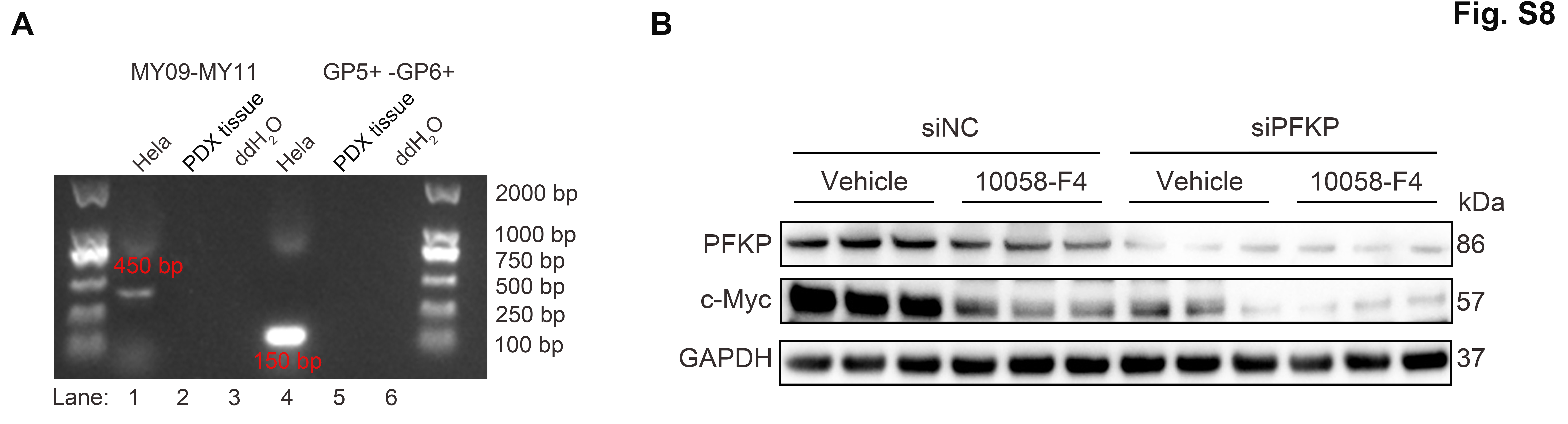

Supplement: Supplementary file 8 — Supplementary Material 8. The co-treatment of both PFKP inhibition and 10058-F4 has inhibitory effect on growth in PDX models. (A) Gel electrophoresis image showing PCR amplification products using primer pairs MY09/MY11 and GP5+/GP6+, testing for HPV DNA in Hela cells (positive control), PDX tumor samples, and ddH2O (negative control). (B) Tumor tissues derived from each group were subjected to immunoblotting. [file 12943_2024_2051_MOESM8_ESM.png]

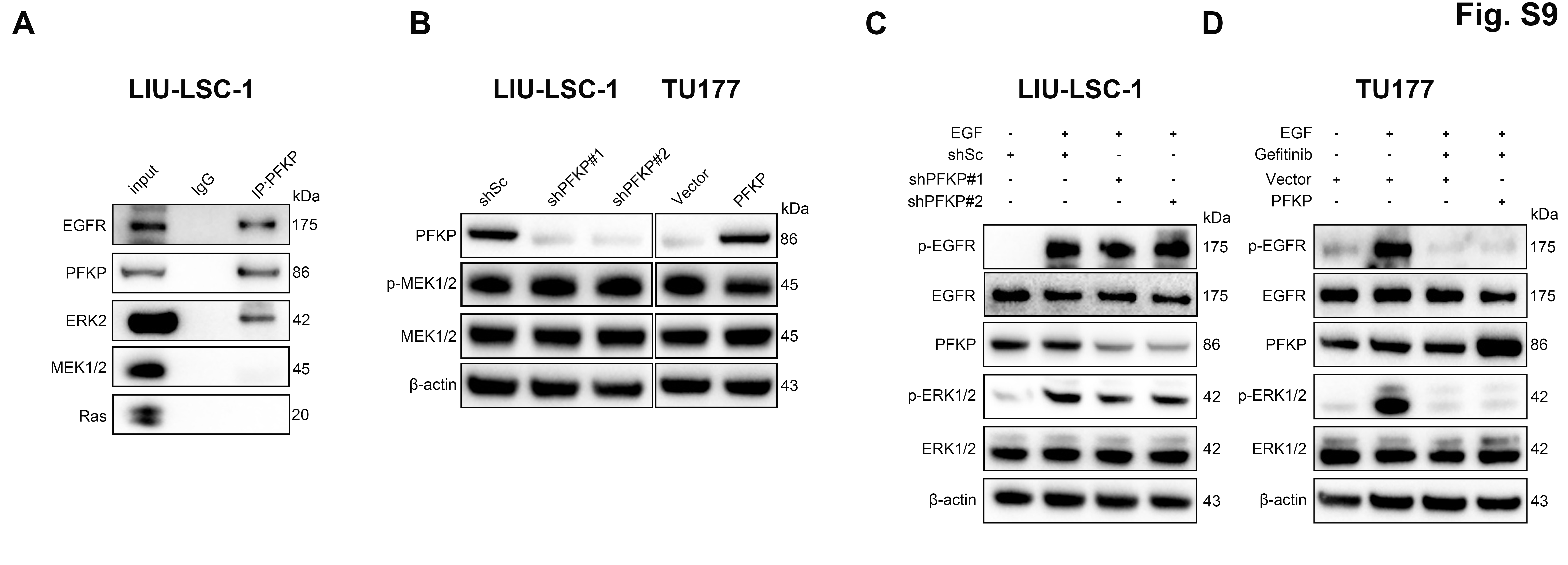

Supplement: Supplementary file 9 — Supplementary Material 9. PFKP is involved in EGFR-mediated activation of ERK1/2. (A) immunoprecipitation (IP) assay followed by Western blotting analysis showing the interaction between PFKP and components of the EGFR-ERK1/2 signaling pathway, including EGFR, ERK2, MEK1/2, and RAS in LIU-LSC-1 cells. (B) Western blotting was used to detect the expression of the indicated proteins after knocking down PFKP in LIU-LSC-1 cells or overexpressing PFKP in TU177 cells. (C) LIU-LSC-1 cells transfected with PFKP shRNA were stimulated with or without EGF (50 ng/mL) for 15 min. (D) Western blotting analysis assesses the effects of PFKP overexpression and the application of the EGFR inhibitor Gefitinib (10 ?M for 48 hours) on EGFR and ERK1/2 activation in TU177 cells, and EGF treatment (50 ng/mL for 1 hour). [file 12943_2024_2051_MOESM9_ESM.png]

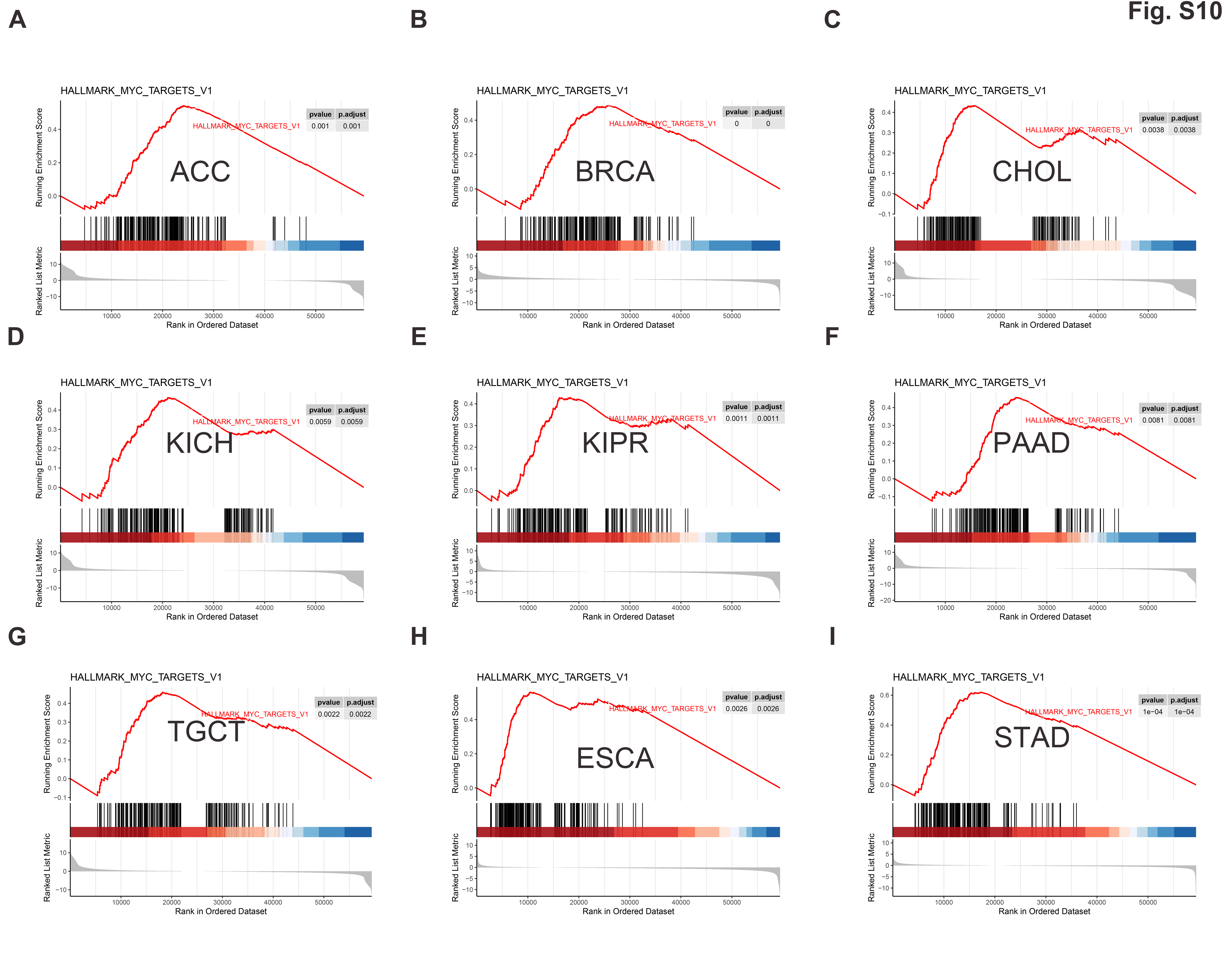

Supplement: Supplementary file 10 — Supplementary Material 10. PFKP expression positively correlates with the activation of the HALLMARK_MYC_TARGET pathway in multiple human cancer tissues. Gene set enrichment analysis (GSEA) was performed to reveal the association between PFKP and the activation of the HALLMARK_MYC_TARGET pathway. (A) Adrenocortical Carcinoma (B) Breast invasive carcinoma (C) Cholangiocarcinoma (D) Kidney Chromophobe (E) Kidney renal papillary cell carcinoma (F) Pancreatic adenocarcinoma (G) Testicular Germ Cell Tumors (H) Esophageal carcinoma (I) Stomach adenocarcinoma. [file 12943_2024_2051_MOESM10_ESM.png]

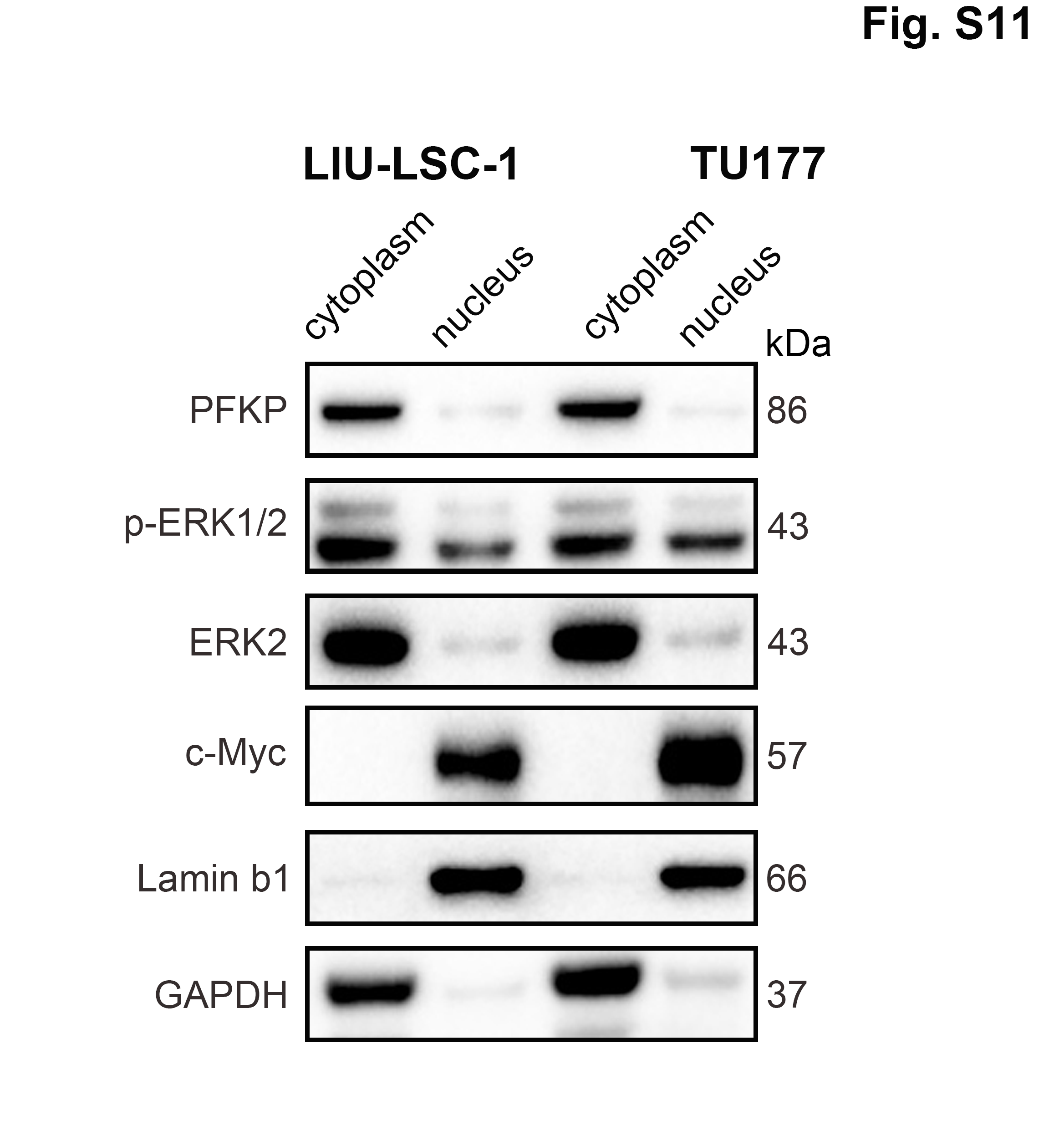

Supplement: Supplementary file 11 — Supplementary Material 11. Western blotting analysis detects the subcellular localization of PFKP, phosphorylated ERK1/2 (p-ERK1/2), ERK2, and c-Myc in the cytoplasmic and nuclear fractions of LIU-LSC-1 and TU177 cells. Lamin b1 and GAPDH are included as nuclear and cytoplasmic markers, respectively, to confirm the purity of the fractions. [file 12943_2024_2051_MOESM11_ESM.png]

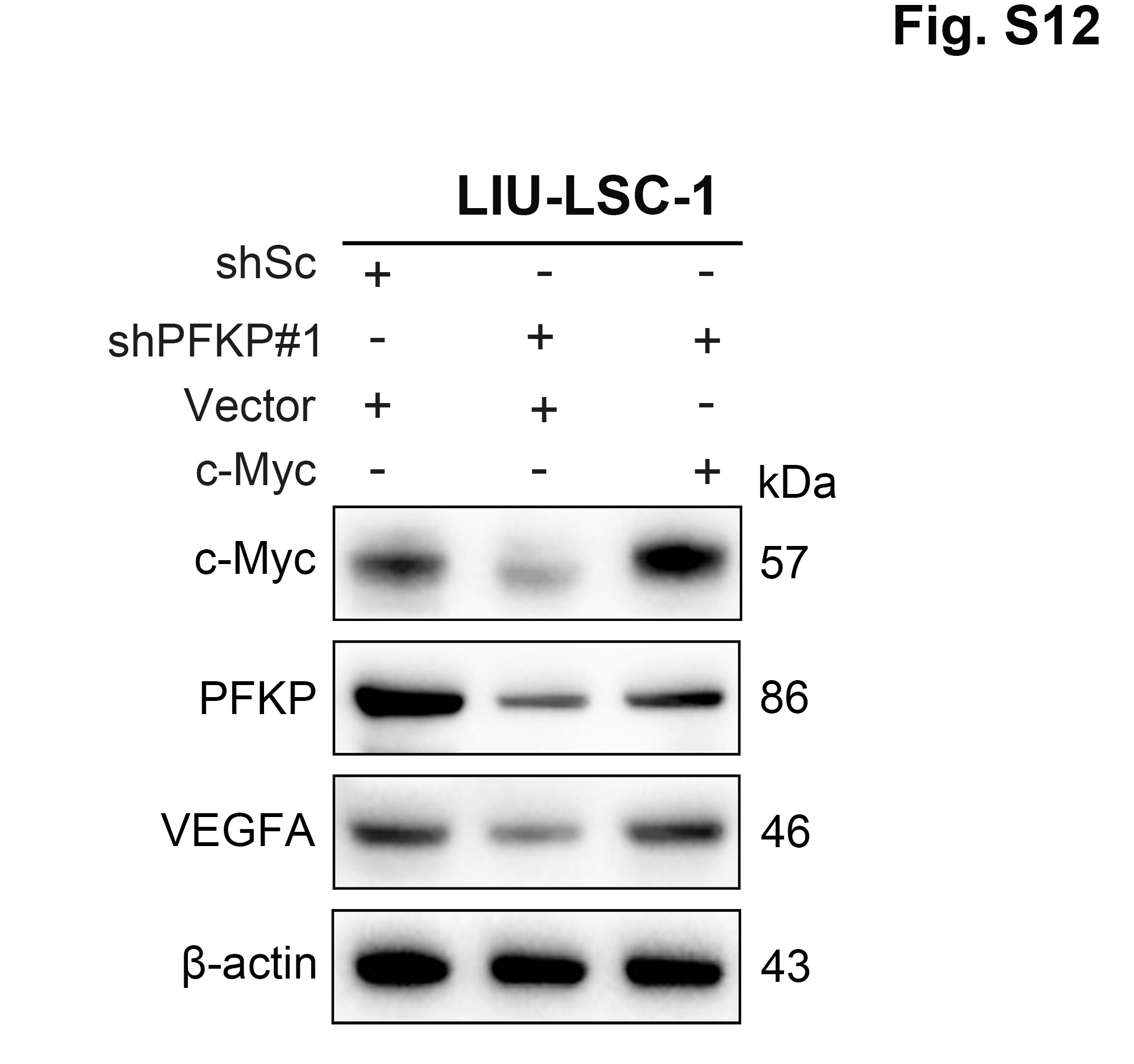

Supplement: Supplementary file 12 — Supplementary Material 12. Cells were transfected with shRNA specific for PFKP (shPFKP#1) or a scramble control (shSc), and with or without a c-Myc expression vector. The presence of c-Myc, PFKP, and VEGFA proteins was detected using Western blotting. [file 12943_2024_2051_MOESM12_ESM.png]

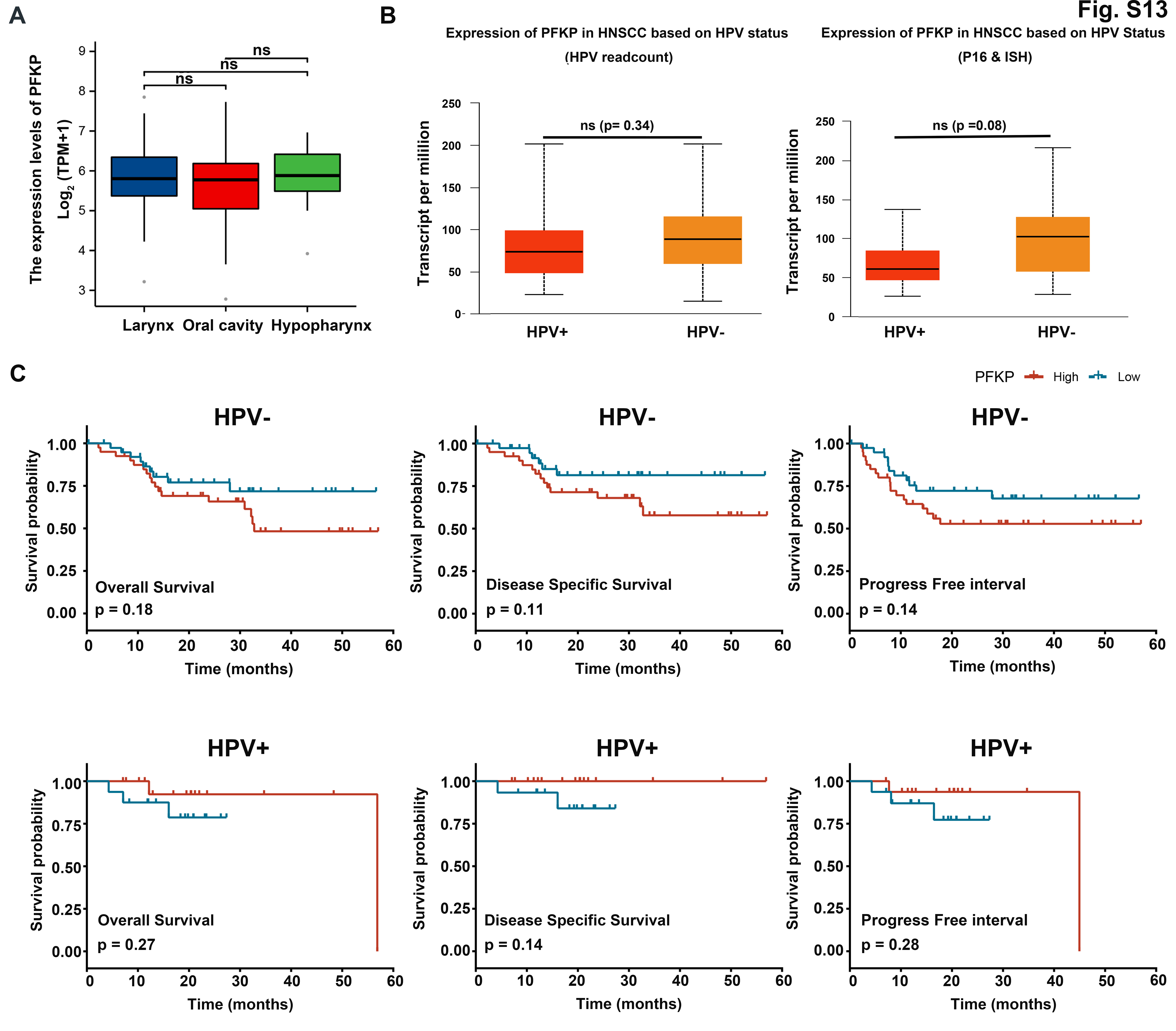

Supplement: Supplementary file 13 — Supplementary Material 13. Analysis of PFKP expression in HNSCC across various anatomical locations and HPV status, and its association with patient survival outcomes in TCGA dataset. (A). Expression levels of the PFKP gene in three different anatomical locations of HNSCC (larynx, oral cavity, and hypopharynx cancers). (B). Expression of PFKP in HNSCC based on HPV status. Data source: UALCAN website (https://ualcan.path.uab.edu/). (C). Kaplan-Meier survival curves for HNSCC patients based on HPV status and PFKP expression levels. The top row displays OS, DSS, and PFI, respectively, for HPV- patients (80 cases), and the bottom row shows the same metrics for HPV+ patients (33 cases). [file 12943_2024_2051_MOESM13_ESM.png]
