# Supplementary material for: A positive feedback loop between PFKP and c-Myc drives head and neck squamous cell carcinoma progression
Source: Mol Cancer. 2024 Jul 9;23:141. doi: 10.1186/s12943-024-02051-6 (PMC11232239; doi:10.1186/s12943-024-02051-6)
Supplement: Supplementary file 14 — Supplementary Material 14: : Supplementary Table 1. Clinical features of 120 HNSCC patients. [file 12943_2024_2051_MOESM14_ESM.docx]

**Supplementary Table 1.** Clinical features of 120 HNSCC patients

| Characteristics | Number of Cases（%) |
| --- | --- |
| Age |  |
| < 60 | 49(40.8) |
| ≥ 60 | 71(59.2) |
| Gender |  |
| Female | 10(8.33) |
| Male | 110(91.7) |
| T Stage |  |
| T1 | 35(29.1) |
| T2 | 24(20.0) |
| T3 | 34(28.3) |
| T4 | 27(22.5) |
| lymph node metastasis |  |
| N0 | 87(72.5) |
| N+ | 33(27.5) |
| Distant metastasis |  |
| M0 | 120(100) |
| M1 | 0(0) |
| Tumor location |  |
| Hypopharynx | 46(38.3) |
| Larynx | 74(61.7) |

­

**Supplementary Table 2.** Clinical characteristics of donor patients

| Clinical characteristics of PDO donor patients | | | | | | |  |
| --- | --- | --- | --- | --- | --- | --- | --- |
| NO. | Cancer type | Age | Gender | T stage | Cancer lymph node metastasis | Distant metastasis | HPV  Status |
| Patient 1 | Hypopharyngeal squamous cell carcinoma | 56 | Male | T3 | N1 | M0 | negative |
| Patient 2 | Laryngeal squamous cell carcinoma | 60 | Male | T3 | N1 | M0 | negative |

| Clinical characteristics of PDX donor patient | | | | | | |  |
| --- | --- | --- | --- | --- | --- | --- | --- |
| NO. | Cancer type | Age | Gender | T stage | Cancer lymph node ­­­metastasis | Distant metastasis | HPV  Status |
| Patient 1 | Laryngeal squamous cell carcinoma | 59 | Male | T3 | N1 | M0 | negative |

**Supplementary Table 3.** Detailed information of antibodies

| **Antibodies** | **Source** | **Application** |
| --- | --- | --- |
| PFKP | Cell Signaling Technology (Cat#12746) | 1:100 for IHC; 1:1000 for WB; 1:50 for IP; 1:200 for IF |
| PFKP | Santa Cruz (Cat #Sc-514824) | 1:500 for WB |
| GAPDH | Cell Signaling Technology (Cat #2118) | 1:1000 for WB |
| β-actin | Sigma-Aldrich (Cat #A1978) | 1:1000 for WB |
| P63 | Proteintech (Cat #12143-AP) | 1:200 for IHC |
| CK13 | Proteintech (Cat #66684-1-lg) | 1:1000 for IHC |
| Ki-67 | Cell Signaling Technology (Cat #9449) | 1:800 for IHC; 1:2000 for IF |
| CD31 | Abcam (Cat #ab76533) | 1:100 for IHC |
| N-cadherin | Proteintech (Cat #66219-1-lg) | 1:5000 for WB |
| Snail | Cell Signaling Technology (Cat #3879) | 1:1000 for WB |
| Vimentin | Santa Cruz (Cat #sc-53464) | 1:200 for WB |
| VEGFA | Proteintech (Cat #19003-1-AP) | 1:2000 for WB |
| Phospho-ERK1/2 (Thr202/Tyr204) | Cell Signaling Technology (Cat #4370) | 1:2000 for WB |
| ERK1/2 | Cell Signaling Technology (Cat #9102) | 1:1000 for WB |
| ERK2 | Abcam (Cat #ab227134) | 1:100 for WB; 1:100 for IP |
| Flag | Cell Signaling Technology (Cat #8146) | 1:1000 for WB; 1:50 for IP |
| HA | Cell Signaling Technology (Cat #3724) | 1:1000 for WB; 1:50 for IP |
| c-Myc | Abcam (Cat #ab32072) | 1:1000 for WB; 1:100 for IF; 1:100 for IHC |
| c-Myc(phospho S62) | Abcam (Cat #ab185656) | 1:1000 for WB |
| Ub | Cell Signaling Technology (Cat #20326) | 1:1000 for WB |
| Lamin B1 | Proteintech (Cat #12987-1-AP) | 1:5000 for WB |
| EGFR | Cell Signaling Technology (Cat #4267) | 1:1000 for WB |
| Phospho-EGFR (Tyr1068) | Cell Signaling Technology (Cat #3777) | 1:1000 for WB |
| MEK1/2 | Proteintech (Cat #11049-1-AP) | 1:5000 for WB |
| Phospho-MEK1/2 (Ser217/221) | Cell Signaling Technology (Cat #9154) | 1:1000 for WB |
| RAS | Proteintech (Cat #81615-1-RR) | 1:20000 for WB |

**Supplementary Table 4**. Primer information for PCR analysis

| **Prime name** | **Primer sequence (5'-3')** |
| --- | --- |
| β-actin FORWARD | CTGGCACCACACCTTCTACAATG |
| β-actin REVERSE | GGCGTACAGGGATAGCACAG |
| PFKP FORWARD | CGGAAGTTCCTGGAGCACCTCTC |
| PFKP REVERSE | AAGTACACCTTGGCCCCCACGTA |
| c-Myc FORWARD | TCAAGAGGCGAACACACAAC |
| c-Myc REVERSE  MY09  MY11  GP5+  GP6+ | GGCCTTTTCATTGTTTTCCA  CGT CCM ARR GGA WAC TGATC′  GCM CAG GGW CAT AAY AAT GG  TTTGTTACTGTGGTAGATACTAC  CTTATACTAAATGTCAAATAAAAA |

**Supplementary Table 8.** Oligonucleotide sequences used in this study

| **Note** | **Sequences (5'-3')** |
| --- | --- |
| shSc | TTCTCCGAACGTGTCACGT |
| shPFKP#1 | GGCTGAAGGAGCAATTGAT |
| shPFKP#2 | GCTCCATTCTTGGGACAAA |
| shc-Myc#1 | CACCTATGAACTTGTTTCA |
| shc-Myc#2 | GGTCAGAGTCTGGATCACC |

**Supplementary Table 9.** Primers used for luciferase reporter in this study

|  | **Gene** | **Forward primers (5'-3')** | **Reverse primers (5'-3')** |
| --- | --- | --- | --- |
| Wild-type | PFKP | CCCCAGCCCTTACATACAGACAAAC | CATCGCCTTCGCCCGGTTCT |
| Mutated | Site 1 | CACTTACACGgcatCGTGCGCACATGC | TGTGTGTGAGTGTGCACA |
| Mutated | Site 2 | CCTCCGCGTCgctcTGGGGTCTCCGCGCCCCCATTCCAC | CTCGCGGCAGGCCCGGCC |

**Supplementary Table 10.** Primers used for CHIP Assays in this study

|  | **Sense (5'-3')** | **Antisense (5'-3')** |
| --- | --- | --- |
| Site #1  (PCR) | TAAGCAAAAGAAAGGAAATAAA | CAAACAAATAAATGAAAAGGCA |
| Site #2  (PCR) | AGCGCCGCCCCAGGAGGAAT | CGATGGCGGGGAGGGGTGGA |
| NBR  (qRT-PCR) | GTCGCCTGGGCACTCTTTC | AGGCCCTCTGAGTACATCTGG |
| PBR  (qRT-PCR) | TGCTCAGAAACATTTACGTG | GCTGTTACCACTACGAAGATA |
